# A Rational Model of Incremental Argument Interpretation: The Comprehension of Swedish Transitive Clauses

**DOI:** 10.3389/fpsyg.2021.674202

**Published:** 2021-10-15

**Authors:** Thomas Hörberg, T. Florian Jaeger

**Affiliations:** ^1^Department of Linguistics, Stockholm University, Stockholm, Sweden; ^2^Department of Computational Science and Technology, KTH Royal Institute of Technology, Stockholm, Sweden; ^3^Department of Brain and Cognitive Sciences, University of Rochester, Rochester, NY, United States; ^4^Department of Computer Science, University of Rochester, Rochester, NY, United States

**Keywords:** language comprehension, argument interpretation, grammatical function assignment, expectation-based processing, Bayesian inference, self-paced reading, Swedish

## Abstract

A central component of sentence understanding is verb-argument interpretation, determining how the referents in the sentence are related to the events or states expressed by the verb. Previous work has found that comprehenders change their argument interpretations incrementally as the sentence unfolds, based on morphosyntactic (e.g., case, agreement), lexico-semantic (e.g., animacy, verb-argument fit), and discourse cues (e.g., givenness). However, it is still unknown whether these cues have a privileged role in language processing, or whether their effects on argument interpretation originate in implicit expectations based on the joint distribution of these cues with argument assignments experienced in previous language input. We compare the former, *linguistic* account against the latter, *expectation-based* account, using data from production and comprehension of transitive clauses in Swedish. Based on a large corpus of Swedish, we develop a rational (Bayesian) model of incremental argument interpretation. This model predicts the processing difficulty experienced at different points in the sentence as a function of the Bayesian surprise associated with changes in expectations over possible argument interpretations. We then test the model against reading times from a self-paced reading experiment on Swedish. We find Bayesian surprise to be a significant predictor of reading times, complementing effects of word surprisal. Bayesian surprise also captures the qualitative effects of morpho-syntactic and lexico-semantic cues. Additional model comparisons find that it—with a single degree of freedom—captures much, if not all, of the effects associated with these cues. This suggests that the effects of form- and meaning-based cues to argument interpretation are mediated through expectation-based processing.

## Introduction

Language understanding requires comprehenders to integrate incoming information to form hypotheses about the intended structure and meaning of sentences. One of the central components of this process is argument interpretation: determining how the referents of the verb’s arguments relate to the events or states expressed by the verb. This determines, for example, whether an argument refers to the *actor* of the event described by the verb, i.e., the most agent-like referent, or the *undergoer* of that event, i.e., the most patient-like referent (see e.g., Dowty, 1991; Primus, 2006). This way, argument interpretation informs us about *who did what to whom*.^1^ This interpretation proceeds incrementally, with comprehenders changing their hypotheses about the intended argument role assignment as the sentence unfolds and more information becomes available. For example, upon hearing a sentence starting with “The policeman …”, the policeman might initially be interpreted as the likely actor of an event to be described. This interpretation will change if the next words are “… was arrested …”. Previous work has found that incremental argument interpretation is affected by a wide range of linguistic cues. This includes both form-based (e.g., case-making) and meaning- or discourse-based properties of the arguments (e.g., animacy, givenness), as well their interactions with verb semantics (e.g., Ferreira and Clifton, 1986; MacWhinney and Bates, 1989; MacDonald et al., 1994; Trueswell et al., 1994; McRae et al., 1998; Kamide et al., 2003; Gennari and MacDonald, 2008; Bornkessel-Schlesewsky and Schlesewsky, 2009; Wu et al., 2010).

While the effects of these cues are now well-attested, questions remain about their theoretical interpretation. Some accounts attribute a privileged role to argument properties that have been linked to increased “accessibility” of argument’s referents in memory (Bornkessel and Schlesewsky, 2006; Kuperberg, 2007; Alday et al., 2014; see also Nakano et al., 2010; Szewczyk and Schriefers, 2011). This includes conceptual (e.g., animacy, number) and discourse-based (e.g., givenness, definiteness) properties of arguments (henceforth *prominence* cues) as well as arguments’ morphosyntactic properties (e.g., case-marking). For example, some accounts consider prominence and morphosyntactic cues to argument interpretation to either be the *only* information that is taken into account during initial stages of processing (Bornkessel and Schlesewsky, 2006), or to be utilized by a separate combinatorial processing stream (Kuperberg, 2007: 37). On these *linguistic* accounts, other information—such as the plausibility of verb-argument combinations—is either taken into account only at a later stage of processing (Bornkessel and Schlesewsky, 2006), or processed in parallel but by other processing mechanisms (Kuperberg, 2007). Competing, expectation-based accounts attribute the effect of prominence and other cues to implicit expectations based on the distribution of cues in previously experienced language input (e.g., MacDonald et al., 1994; Trueswell et al., 1994; McRae et al., 1998; Narayanan and Jurafsky, 1998; Kempe and MacWhinney, 1999; Vosse and Kempen, 2000, 2009; Tily, 2010; MacDonald, 2013; Bornkessel-Schlesewsky and Schlesewsky, 2019; Rabovsky, 2020). Both linguistic and expectation-based accounts predict that prominence and other cues affect incremental argument interpretation. The two types of accounts differ, however, with respect to whether these effects are taken to be direct, or mediated through expectations. Previous work has found that expectation-based models provide a good fit against human data: across a variety of different structural contexts, expectation-based models correctly predict in which sentences, and where in those sentences, comprehenders will experience processing difficulty (e.g., Demberg and Keller, 2008; Levy, 2008; Boston et al., 2011; Frank and Bod, 2011; Frank et al., 2015). This includes—sometimes complex—interactions between cues that require additional explanations under the linguistic account (we provide examples in Section “Previous Work on Argument interpretation”), as well as qualitative differences in the effects of the same cue across languages (MacWhinney et al., 1984; MacWhinney and Bates, 1989; Desmet et al., 2002, 2006; Acuña-Fariña et al., 2009). This ability to correctly predict the data is particularly noteworthy since the expectation-based account is more parsimonious than the linguistic account: the expectation-based account allows linguistic cues to affect argument interpretation only to the extent that these cues affect the relative probability of different argument interpretations. Since researchers can determine the latter—the objective probabilities—from appropriate language databases, the expectation-based account has few degrees of freedom in predicting language comprehension. In short, previous work suggests that the expectation-based account provides a parsimonious, unifying explanation for a variety of otherwise puzzling processing behaviors. Direct comparisons to the linguistic account on the same data have, however, been lacking. This is the comparison we aim to provide here.

**FIGURE 1:**
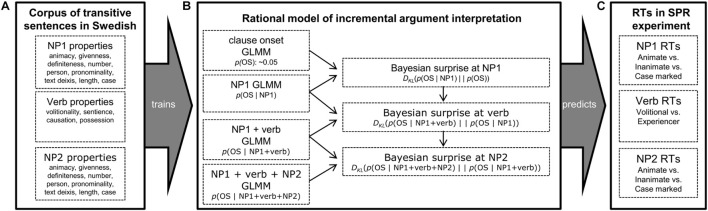
Illustration of the approach taken in the present study. **Panel A:** A corpus of 16,552 transitive clauses in written Swedish is created and annotated for 16 different cues to argument interpretation. **Panel B:** From this corpus, the probability of OS vs. SO order is estimated at four different sentence regions, using all cues available up to that point in the sentence. The Bayesian surprise at each sentence region—quantifying the incremental change in expectations about argument interpretation—is then derived from these probabilities. **Panel C:** The rational model is tested by predicting human reading times at different sentence regions from the model-predicted Bayesian surprise.

Our general approach to this question is illustrated in Figure 1. We develop a *rational expectation-based model* of incremental argument interpretation that links processing times to the Bayesian surprise over changes in argument interpretation as the sentence unfolds. To test this model, we draw on a corpus of transitive clauses in written Swedish (Panel A). The corpus is annotated for a large number of cues previously shown to affect argument interpretation, including morpho-syntactic (e.g., case), syntactic (e.g., clause embedding), prominence (e.g., animacy, definiteness, givenness, deixis) and verb-semantic cues (e.g., volitionality). We then use this corpus to estimate, at different points throughout the sentence, the probability of object-subject (OS) vs. subject-object word order (SO) (Panel B), the former order corresponding to an undergoer-initial interpretation, and the latter to an actor-initial interpretation.^2^ These probabilities are taken to approximate comprehender’s expectations—based on previously experienced input—about the underlying argument assignment at different points in the sentence.

We operationalize the cognitive cost associated with changes in these expectations as *Bayesian surprise* (following Kuperberg and Jaeger, 2016; defined below). Once the model is introduced, we use it to derive predictions about comprehension. We use the rational model to design a moving window self-paced reading experiment over sentence stimuli that are predicted to exhibit a large range of Bayesian surprise across stimuli conditions and sentence regions. We test whether Bayesian surprise—derived from the rational model—provides a good *quantitative* and *qualitative* fit against human reading times from this experiment (Panel C). This brings us to the critical comparison that has been lacking in previous work. We compare the fit of the rational model against that of a much less constrained *linguistic model* that can accommodate any type of functional relation between linguistic cues and reading times. This comparison determines whether the rational model—with its hypothesized linear link between Bayesian surprise and reading times—constitutes a parsimonious theory of incremental argument interpretation, explaining effects of various linguistic properties on argument interpretation with a single degree of freedom (the linear effect of Bayesian surprise on RTs). Finally, we investigate how the effects of Bayesian surprise—capturing changes in expectations about *argument interpretation*—relate to effects of word surprisal—an estimate of expectations about *individual words* previously found to be a strong predictor of reading times (e.g., Levy, 2008; Frank and Bod, 2011; Smith and Levy, 2013).

### Previous Work on Argument Interpretation

Previous support for an expectation-based account of argument interpretation has come from studies highlighting how the effects of and interaction between various cues on argument interpretation *qualitatively* match to the distribution of those cues in language use.

This tendency is perhaps most thoroughly attested with regard to the linguistic properties of NPs: linguistic properties that make NP arguments less likely to carry the intended argument assignment also tend to negatively affect processing, compared to linguistic properties that make NP arguments expected candidates for the argument assignment. For example, grammatical subjects are cross-linguistically more frequently animate, definite, 1st/2nd person, pronominal and given (i.e., high in prominence), while objects are more commonly inanimate, indefinite, 3rd person, lexical and new (i.e., low in prominence; e.g., in Dutch: Bouma, 2008; Swedish: Dahl and Fraurud, 1996; Dahl, 2000; German: Kempen and Harbusch, 2004; Norwegian: Øvrelid, 2004; for review, see Du Bois, 2003).^3^ And, when given an implicit choice, speakers preferentially encode animate and previously mentioned referents as subject, rather than object (e.g., English: Bock and Irwin, 1980; Bock and Warren, 1985; German: Nice and Dietrich, 2003; Greek: Feleki and Branigan, 1999; Japanese: Ferreira and Yoshita, 2003; Tanaka et al., 2011; Tagalog: Sauppe, 2017; Chinese: Hsiao and MacDonald, 2016; for a cross-linguistic review, see Jaeger and Norcliffe, 2009). Prominence properties are thus statistically informative about argument assignment, so that expectation-based accounts predict that prominence properties should affect argument interpretation. In line with these qualitative predictions, subject arguments that are low in prominence (e.g., inanimate), and object arguments that are high in prominence (e.g., definite)—and thus unexpected—tend to cause processing difficulty (Kuperberg et al., 2003; Roehm et al., 2004; Philipp et al., 2008; Nakano et al., 2010; Paczynski and Kuperberg, 2011; Muralikrishnan et al., 2015; Czypionka et al., 2017; Philipp et al., 2017). Similarly, structures that are locally ambiguous with respect to argument functions are easier to process when the arguments are prototypical in animacy or referentiality (e.g., reduced relative clauses: Just and Carpenter, 1992; Trueswell et al., 1994; object-relative clauses: Weckerly and Kutas, 1999; Warren and Gibson, 2002; Traxler et al., 2005; Mak et al., 2006, 2008; Gennari and MacDonald, 2008; Hsiao and MacDonald, 2016; temporarily ambiguous transitive sentences: Kaiser and Trueswell, 2004; Frenzel et al., 2011; Kretzschmar et al., 2012).

Another domain for which this parallelism between patterns in the input and processing is now well-documented is the interaction between verb semantics and NP properties. For example, verbs of cognition and perception, expressing private knowledge and subjective experiences (e.g., *know*, *think*, *see*, or *feel*) and volitional verbs, referring to acts that are based upon intentions of an agent (e.g., *avoid*, *choose*, *steal*, or *seek*), most often require an actor referent that is sentient and/or volitionally acting, and therefore animate. The information that prominence cues carry about argument interpretation therefore to some extent depends on the semantics of the verb. Expectation-based accounts thus predict that comprehenders should take the interplay between NP properties and verb semantics into account during argument interpretation. Research on sentence processing suggests that this is indeed the case: NP arguments with prominence or other semantic properties that are unexpected based on the verb’s semantics (Wang et al., 2020; see also Szewczyk and Schriefers, 2013) or that violate the verb’s selectional restrictions result in neural signatures that reflect processing costs (e.g., as in *At the homestead the farmer penalized the ^∗^meadow for laziness*, Kuperberg et al., 2003; Kim and Osterhout, 2005; van Herten et al., 2005, 2006; Kuperberg et al., 2006, 2007; Bornkessel-Schlesewsky et al., 2011; Paczynski and Kuperberg, 2011, 2012). At the same time, comprehension is facilitated when an NP argument is compatible with the semantic role assigned to it by the verb (e.g., in terms of its animacy, Czypionka et al., 2017; Philipp et al., 2017; or in terms of thematic fit, e.g., Trueswell et al., 1994; Garnsey et al., 1997; McRae et al., 1998).

For additional examples and discussion, we refer to Hörberg (2016). This review of the literature came to the conclusion that expectation-based accounts can in most cases explain the effects of cues to argument interpretation. As compelling as these results might be, however, they do not show whether expectations are sufficient to predict the effects of linguistics cues on argument interpretation.

This caveat also applies to previous computational modeling of argument interpretation: pioneering work showed that competition models trained on the statistical relations between linguistic cues and argument assignment can predict the qualitative patterning of, for example, reading times or eye-movements (e.g., MacDonald et al., 1994; Tabor et al., 1997; McRae et al., 1998; Spivey-Knowlton and Tanenhaus, 1998; Vosse and Kempen, 2000, 2009). The goodness of fit of these expectation-based models was not, however, compared against linguistic models that are *not* constrained by the statistics of the input. It is therefore still unclear how much of the variability in reading times associated with linguistic cues can be reduced to expectations. This is the question we seek to address here.

## A Rational Model of Incremental Argument Interpretation

We follow previous expectation-based models of sentence processing and assume that comprehenders incrementally update their implicit expectations about the underlying sentence interpretation as new input becomes available (Jurafsky, 1996; Narayanan and Jurafsky, 1998; Crocker and Brants, 2000; Hale, 2001; Levy, 2008). In rational expectation-based models, sentence interpretation involves continuously shifting from a prior to a posterior probability distribution over possible parses, a process known as *Bayesian belief-updating*. The processing cost associated with new input is in part determined by the amount of new information provided by the input—specifically, the degree of shift in expectations or beliefs about the underlying parse (Levy, 2008, 2011). Formally, this shift can be quantified in terms of Bayesian surprise. Bayesian surprise constitutes a principled measure of the prediction error experienced while processing new input (for review, Friston, 2010) and has been linked to attention (Itti and Baldi, 2009) and learning (Ranganath and Rainer, 2003). More recently, it has been proposed to reflect the amount of information gain at a specific level of linguistic representation incurred while processing new input (Kuperberg and Jaeger, 2016; Yan et al., 2017; for a related approach, see Rabovsky et al., 2018).

Bayesian surprise is equivalent to the Kullback-Leibler (KL) divergence of the posterior distribution with respect to the prior distribution. The KL divergence of probability distribution Q from probability distribution P is defined as:


(1) $$D_{KL}\left(P||Q\right)=\sum_{i}\log_{2}\left(\frac{P\left(i\right)}{Q\left(i\right)}\right)P\left(i\right)$$

The Bayesian surprise of encountering word *w*_*i*_ is therefore equal to the KL divergence between the posterior probability distribution over possible argument role assignment s*ARA* after seeing *w*_*i*_ and the prior distribution of argument role assignments just prior to that on *w*_*i*__–__1_:


(2) $$D_{KL}\left(p\left(ARA|w_{1}\ldots w_{i}\right)||p\left(ARA|w_{1}\ldots w_{i-1}\right)\right)$$

To calculate the Bayesian surprise of a word, or sequence of words, it is necessary to estimate the relevant prior and posterior probability distributions. This can be done by estimating the relevant distributions from corpus data. Previous rational models have, for example, integrated lexical ngram contexts (e.g., Smith and Levy, 2013; Frank et al., 2015), syntactic (Hale, 2001; Levy, 2008, 2011; Linzen and Jaeger, 2014), or other latent structure (Frank and Haselager, 2006; Frank and Yang, 2018). These models have been found to predict word- or region-based reading times (e.g., Demberg and Keller, 2008; Roark et al., 2009; Boston et al., 2011; Frank and Bod, 2011; Smith and Levy, 2013; Linzen and Jaeger, 2014; Brothers and Kuperberg, 2021) or neural indices of processing costs (e.g., Frank et al., 2015; Willems et al., 2016; Rabovsky et al., 2018; Weissbart et al., 2020; Yan and Jaeger, 2020). The rational model presented here differs from those models in that it is intended to quantify the cognitive cost associated with specifically *argument interpretation*. We thus estimate the incremental Bayesian surprise caused by changes in the relative probability of different argument interpretations. We estimate these probabilities based on the corpus statistics of the types of cues found in previous work to affect argument interpretation.

The present focus on argument interpretation is shared with classic competition models (MacDonald et al., 1994; Tabor et al., 1997; McRae et al., 1998; Spivey-Knowlton and Tanenhaus, 1998; Vosse and Kempen, 2000, 2009; MacDonald and Seidenberg, 2006). In these models, processing cost is a function of the agreement between the relative change in activation of competing argument interpretations from one sentence region to another. This is conceptually closely related to Bayesian surprise, which measures the change in the relative support for competing interpretations. Compared to competition models, however, the rational model presented here is functionally less flexible, making it more parsimonious. Whereas competition models allow non-linear relations between changes in activation and RTs (e.g., mediated through the decision threshold, Δ_*crit*_, in McRae et al., 1998), we assume that Bayesian surprise is a linear predictor of reading times (cf. the linear link between word surprisal and RTs demonstrated in Smith and Levy, 2013; but see Brothers and Kuperberg, 2021). This arguably makes the rational model an even stronger test of the expectations-based hypothesis.

We test the rational model against data from the reading of simple transitive clauses in Swedish. In such clauses, information regarding argument role assignment is provided by the grammatical functions of the NP arguments. The subject NP refers to the actor of the event and the object NP to the undergoer of the event. Argument interpretation in such sentences is thus equivalent to the assignment of grammatical functions. We specifically focus on canonical Swedish transitive clauses with subject-object (SO) order, and object-initial sentences with object-verb-subject (OS) order (see Hörberg, 2018). We make the simplifying assumption that comprehenders know—or at least strongly expect—that the sentence they are processing are a transitive clause. For the experiment we present below to test the model, this assumption is plausibly warranted since *all* sentences in the experiment are simple transitive clauses. Previous work has found that comprehenders are sensitive to the distribution of syntactic structures in experiments (e.g., Kaschak and Glenberg, 2004; Fine et al., 2013; Yan and Jaeger, 2020; but see also Harrington Stack et al., 2018). Under this simplifying assumption, the Bayesian surprise over argument interpretations associated with the processing of information available at constituent *C*_*i*_ is:^4^


(3) $$D_{KL}\left(p\left(\mathrm{OS}|C_{1}\ldots C_{i}\right)||p\left(\mathrm{OS}|C_{1}\ldots C_{i-1}\right)\right)$$

The Bayesian surprise in Eq. 3 captures the change in expectations about argument interpretation—specifically, whether the first or the second NP is the subject—based on the cues available in constituent *C*_*i*_ (e.g., the second noun phrase, NP2) with respect to the cues available at the previous constituent *C*_*i*__–__1_ (e.g., NP1 and the verb). Here, we test whether this Bayesian surprise predicts the incremental processing difficulty associated with argument assignment during the comprehension of Swedish transitive sentences.

### Corpus Data

The rational model is trained on a corpus of 16,552 transitive sentences (Panel A in Figure 1) from the Svensk Trädbank treebank (Nivre and Megyesi, 2007). This corpus consists of about 1.3 million words of syntactically annotated Swedish texts from various genres (a subset of the 13 billion word Korp collection, Borin et al., 2012). As described in more detail in the Supplementary Section 1, these sentences display a broad range of structural variation. They consist of canonical transitive sentences with SVO order, object-initial transitive sentences with OVS order, and adverbial-initial sentences with VSO or VOS order. They further vary with respect to NP length, number of auxiliary verbs, verb particles, and adverbials, etc. These sentences were annotated for morphosyntactic (e.g., case-marking, auxiliary verbs), syntactic (embedding, verb-initial vs. verb-medial word order), prominence (e.g., animacy, person, givenness, definiteness), and verb semantic cues (e.g., volitionality, sentience). In total, we annotated 16 different cues, each with two or more possible values (for a full list, see Table 1 and Supplementary Section 1.3). The annotated corpus data is available at https://osf.io/rw5nf/.

**TABLE 1:** All linguistic cues used to predict OS vs. SO order at four different points in the sentence through separate Bayesian mixed-effects regressions (GLMMs).

	Cue	GLMM model		
		NP1 (12 DFs)	NP1 + verb (22 DFs)	NP1 + verb + NP2 (36 DFs)
NP1	animacy (*animate* vs. *inanimate*)	×	×	×
	givenness (*given* vs. *new*)	×	×	×
	definiteness (*definite* vs. *indefinite*)	×	×	×
	number (*singular* vs. *plural*)	×	×	×
	person/egophoricity (*1st* and *2nd person* vs. *3rd person*)	×	×	×
	pronominal (*pronominal* vs. *lexical*)	×	×	×
	case (*unmarked* vs. *subject* vs. *object*)	×	×	×
	text deixis (*text deictic* vs. *other*)	×	×	×
	length (continuous)	×	×	
Verb	volitional (*volitional* vs. *not*)		×	×
	experiencer (*experiencer* vs. *not*)		×	×
	causative (*causative* vs. *not*)		×	×
	possessive (*possessive* vs. *not*)		×	×
	auxiliary (*auxiliary verb(s)* vs. *not*)		×	×
NP2	animacy (*animate* vs. *inanimate*)			×
	givenness (*given* vs. *new*)			×
	definiteness (*definite* vs. *indefinite*)			×
	number (*singular* vs. *plural*)			×
	person/egophoricity (*1st* and *2nd person* vs. *3rd person*)			×
	pronominal (*pronominal* vs. *lexical*)			×
	case (*unmarked* vs. *subject* vs. *object*)			×
	text deixis (*text deictic* vs. *other*)			×
	length (continuous)			×
Other	embedded (*main* vs. *embedded clause*)	×	×	×
	verb before S and O (*verb-initial* vs. *verb-medial*)	×	×	×
Interactions	animacy × volitional		×	
	animacy × causation		×	×
	person × experiencer		×	×
	givenness × possessive			×
	definiteness × possessive		×	×
	pronominality × possessive			×

*The clause onset GLMM is not shown as it only contained the intercept (and the same random effects as the other three GLMMs). The procedure to determine which interactions of cues to include in the model is described in the Supplementary Section 2.1. The total number of degrees of freedom (DFs) for each GLMM are shown at the top of each column. Text deixis concerns whether an NP is a neuter pronominal or demonstrative object (i.e., det and detta – “that”) that refers back to a proposition in the immediate left context. Objects that consist of such NPs very frequently occupy the sentence initial position Swedish (Hörberg, 2016, 2018). Text deixis thus serves as a highly reliable cue to argument interpretation.*

### Estimating the Distributions of Object-Subject vs. Subject-Object Orders

We use this corpus to estimate the Bayesian surprise at three sentence regions: at NP1, at the verb, and at NP2. These estimates are used below to test whether Bayesian surprise predicts reading times at these different sentence regions. As shown in Figure 1 (Panel B), the Bayesian surprise at these three sentence regions is obtained by estimating the distribution of OS vs. SO order at four different points in the sentence: (i) at the clause onset prior to any sentence input, (ii) after NP1 has been processed, (iii) after NP1 and the verb has been processed, and (iv) after NP1, the verb, and NP2 has been processed. The Bayesian surprise at NP1 is the KL divergence between the distribution of OS vs. SO after NP1 has been processed (ii) and the distribution of OS vs. SO at the clause onset prior to NP1 (i), etc.

These distributions of OS vs. OS order at (i)–(iv) was estimated by fitting four separate Bayesian mixed-effects logistic regressions (GLMMs). Each of these four GLMMs included all annotated cues available up to that point in the sentence. Table 1 summarizes these cues and which GLMM included them. The predictors and why they were chosen are further motivated in the Supplementary Section 1.3 (see also Hörberg, 2016).

The use of Bayesian GLMMs with regularizing priors makes it possible to model both cues with gradient effects on argument interpretation (e.g., definiteness) and cues that are fully disambiguating (e.g., case-marking). Regularizing priors “shrink” coefficient estimates toward zero, thereby reducing the chance of overfitting to the data, and facilitate model convergence. We used somewhat weaker priors than is standardly recommended for *data analysis* (e.g., Gelman, 2006; Gelman et al., 2008). *Post-hoc* analyses presented in the Supplementary Section 2.3, confirmed that our results do not change over a large range of prior strengths. For the intercept, we used a normal prior with mean −2.994 (the log-odds of the overall proportion of OS order, which is 0.05), and a scale of 2.5. For all other fixed effects, we used Student *t* prior centered at 0 with 30 degrees of freedom and a scale of 5. For the standard deviation of random effects (i.e., the by-genre intercepts), we use a Cauchy prior with location 0 and scale 2. All models were fit with the statistical package *brms* (Bürkner, 2017, 2018) in *R* (R Core Team, 2020). All analysis scripts are available at https://osf.io/rw5nf/.

The fitted GLMMs provide estimates of the probability of OS vs. SO order for any of the four sentence regions and all 16,552 sentences in our corpus. These estimated probabilities can then be plugged into Eq. 3, yielding the predicted Bayesian surprise for the three sentence regions NP1, verb, and NP2. Without refitting the GLMMs, the same procedure can also be used to calculate the predicted Bayesian surprise for any hypothetical combination of linguistic cues, including combinations that were never observed in the corpus. The NP1 + verb + NP2 GLMM, for example, makes predictions about OS vs. SO order for all 2^36^ hypothetically possible combinations of the 36 predictors in the GLMM (see Supplementary Table 8).

### Illustrating the Model Predictions

To illustrate the predictions of the rational model, we focus on the subset of transitive sentences as well as the subset of NP and verb semantic properties for which the rational model predicts the greatest variation in Bayesian surprise. Predictions for a wider range of structures and properties are presented in the Supplementary Section 3. The qualitative predictions we illustrate here also inform the interpretation of the self-paced reading experiment we present below.

Since Swedish lacks case-marking on nouns, OVS sentences with pronominal subjects are morpho-syntactically ambiguous with respect to argument interpretation until the presentation of the post-verbal subject, which disambiguates the sentences toward OVS. These sentences are a perfect test case for investigating how the expectation for a particular argument interpretation varies as a function of the cues of NP1, the verb, and their interactions. Consider the following example sentences taken from the corpus:


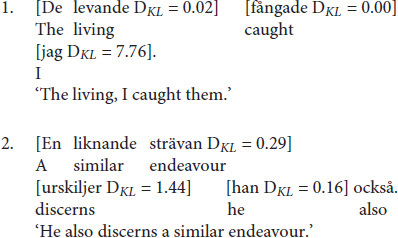


**FIGURE 2:**
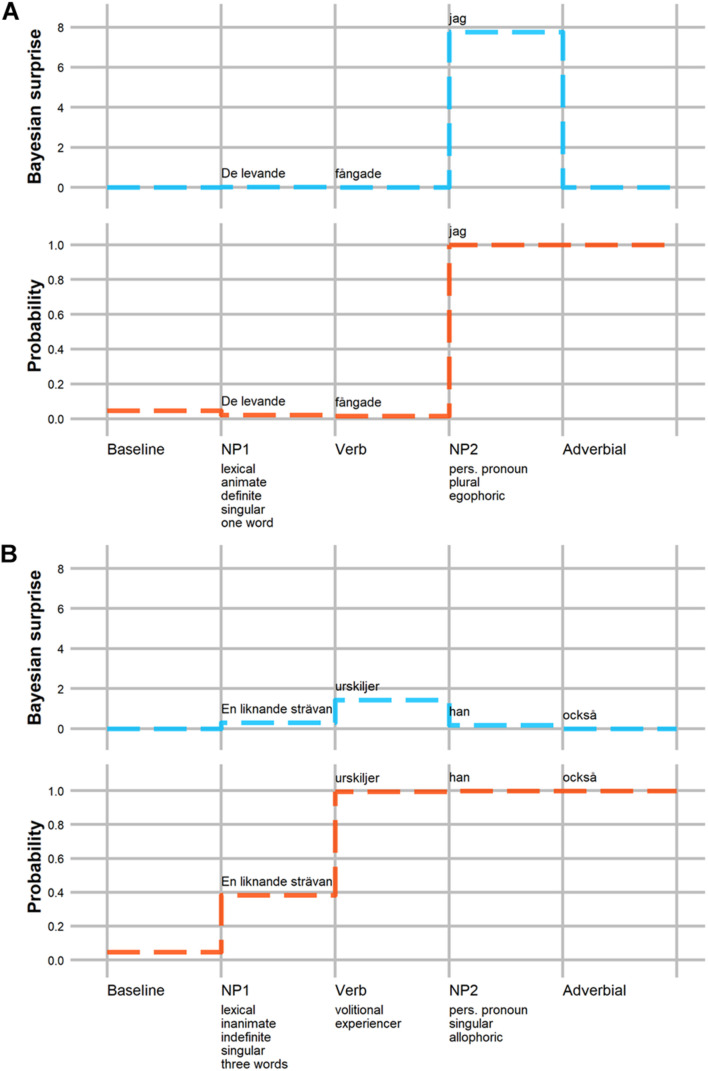
Model-predicted probability of OS order (bottom of each panel) and Bayesian surprise (top) for two example sentences from the corpus. **Panel A:** information that disambiguates the sentence toward OVS order is provided at NP2. **Panel B:** information that speaks in favor of OVS order accrues over the sentence constituents. The relevant cues of each sentence are specified on the x-axis.

Figure 2 (Panel A) illustrates the Bayesian surprise as well as the probability of OS order at each constituent of example (1). Prior to the beginning of the sentence, SO order is much more likely than OS order, *p*(OS) = 0.047. The first NP (*De levande*) in (1) is high in prominence (*De levande* is animate and definite). These cues are predicted to make OS order even less likely after NP1 is processed (*p*(OS) = 0.02). This predicted change in beliefs is, however, small since OS order was unexpected to begin with. As a consequence, Bayesian surprise is close to zero at NP1. Similarly, the semantics of the verb in (1) do not conflict with the strong expectations for SO order either. As a consequence, the probability of an OS order remains low after processing the verb, *p*(OS) = 0.02, and Bayesian surprise on the verb is predicted to be close to zero (*D*_*KL*_ = 0.00). This changes, however, when NP2 (*jag*) is encountered. This NP consists of a personal pronoun with nominative case-marking, providing unambiguous evidence for OS order. The rational model thus predicts a large increase in the probability of OS order, *p*(OS) = 0.99, and correspondingly large Bayesian surprise (*D*_*KL*_ = 7.76).

In example (2), on the other hand, NP1 is low in prominence (*En liknande strävan* is inanimate and indefinite), and therefore provides some initial evidence for an object-initial interpretation, *p*(OS) = 0.38. As illustrated in Figure 2 (Panel B), this is reflected in a small but noticeable increase in Bayesian surprise at NP1 (*D_*KL*_* = 0.29). In (2), the upcoming verb *urskiljer* is both volitional as well as experiencer. In combination with the preceding NP1, these verb semantics strongly bias for an object-initial interpretation, *p*(OS) = 0.99. This large increase in the probability of OS order results in large Bayesian surprise at the verb (*D_*KL*_* = 1.44). In this context, the final NP2 (*han*)—a personal pronoun with nominative case-marking like in (1)—does *not* provide much additional evidence for an object-initial interpretation, *p*(OS) = 0.99. The rational model thus predicts little Bayesian surprise at NP2 (*D_*KL*_* = 0.16).

**FIGURE 3:**
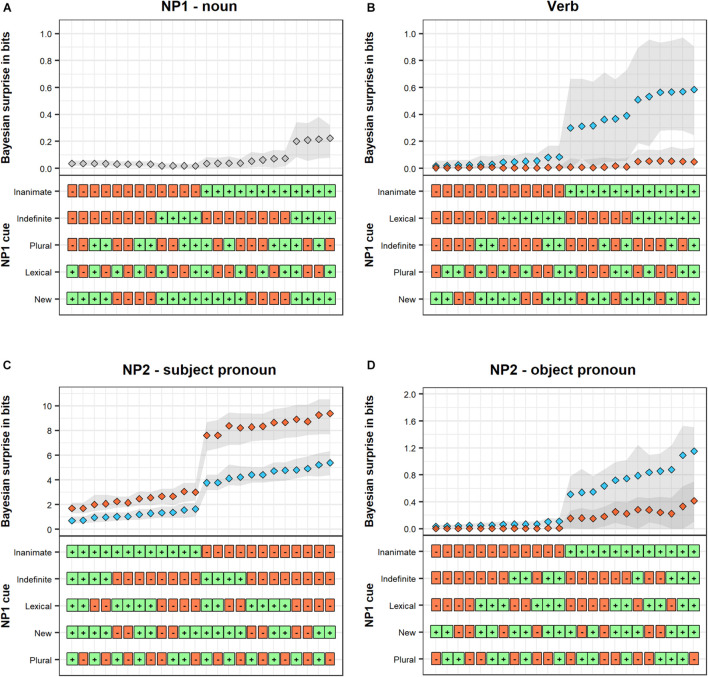
Predicted Bayesian surprise of the NP1, verb, and NP2 constituents of a transitive sentence with a lexical NP1 and a 1st person pronoun NP2. Bayesian surprise is shown as a function of NP1 prominence cues (green square with plus indicates presence of feature) and verb semantics (red for volitional and blue for experience verbs). **Panel A:** Bayesian surprise on NP1 (verb semantic information not yet available). **Panel B:** Bayesian surprise on the verb. **Panel C:** Bayesian surprise for NP2 when NP2 is a case-marked object pronoun disambiguating toward SVO order. **Panel D:** Bayesian surprise for NP2 when NP2 is a case-marked subject pronoun disambiguating toward OVS order. Shaded areas illustrate 89% highest posterior density intervals (HPDIs) of predicted Bayesian surprise, calculated on the basis of the posterior predictions of the underlying GLMMs. Note that the range of the y-axis as well as the order of cues differ between plots. For each panel cues are ordered in decreasing importance from top to bottom.

Figure 3 illustrates the predicted effects of a wider range of cues to argument assignment. It shows changes in Bayesian surprise in sentences with a 3rd person lexical NP1 and a 1st person pronoun NP2 (as in example (1) as a function of NP1 and verb semantic cues. Panels A and B show the Bayesian surprise on NP1 and the verb, respectively. Panels C and D summarize Bayesian surprise on NP2 depending on whether that NP is a subject or object pronoun. The patterns in Figure 3 further confirm that NP prominence cues (animacy, definiteness, number, etc.) interact with verb semantics in determining the probability of OS order, and thus Bayesian surprise. This is visible in Panels B–D, where the difference between the red and blue lines (indicating verb semantics) *strongly* depends on the specific properties of NP1. Also striking is that animacy is the NP1 cue that most strongly interacts with verb semantics. This is evident, for example, in Panel B in a jump in Bayesian surprise for experiencer verbs—but not for volitional verbs—when the preceding NP1 is inanimate, compared to when it is animate. Similarly strong interactions between NP1 animacy and verb semantics are also observed in Panels C-D, though the *direction* of that interaction depends on the case-marking of NP2. Finally, the overall differences between Panels C and D further illustrate how NP2 case-marking affects Bayesian surprise, and how these effects, too, depend on verb semantics (and NP1).

These strong interactions between NP1 animacy, verb semantics, and NP2 case-marking are in line with previous work on subject-object order preferences in Swedish (Rahkonen, 2006; Hörberg, 2018). They are also in line with the observation that animate subjects—in particular 1st/2nd person pronoun subjects—first and foremost occur with experiencer verbs, and secondly with volitional verbs (Dahl, 2000). The information that person (i.e., 1st and 2nd vs. 3rd person) and animacy provide about argument assignment is therefore expected to interact with the semantics of the verb: a 3rd person NP is more predictive of OS order when it co-occurs with an experiencer verb. This is reflected in the strong interplay between NP1 prominence cues and verb semantics, described in more detail below. These patterns of effects motivate the design of the self-paced reading experiment we present next.

## Testing the Predictions of the Rational Model Against Human Reading Times

We test the predictions of the rational model in a self-paced reading experiment against Swedish transitive sentences with either SVO or OVS order. Sentence stimuli were designed to test the predicted main effects and interactions of constituent order, animacy and verb class shown in Figure 3. We chose to manipulate these specific cues—constituent order, animacy, and verb semantics—because we found them to have the strongest effects on Bayesian surprise (for additional details, see Hörberg, 2016). The design of our experiment thus holds constant all other cues to argument assignment listed in Table 1.^5^ It is important to note, however, that the rational models’ predictions are based on *all* cues present in the stimuli, i.e., all properties listed in Table 1. In the context of this experiment, it is thus only constituent order, verb semantics, and animacy that affect the predicted Bayesian surprise. The two questions we seek to address are (1) to what extent the differences in Bayesian surprise across items and sentence regions explain differences in reading times, and (2) whether Bayesian captures most (or even all) of the effects of constituent order, animacy, and verb semantics on RTs.

**FIGURE 4:**
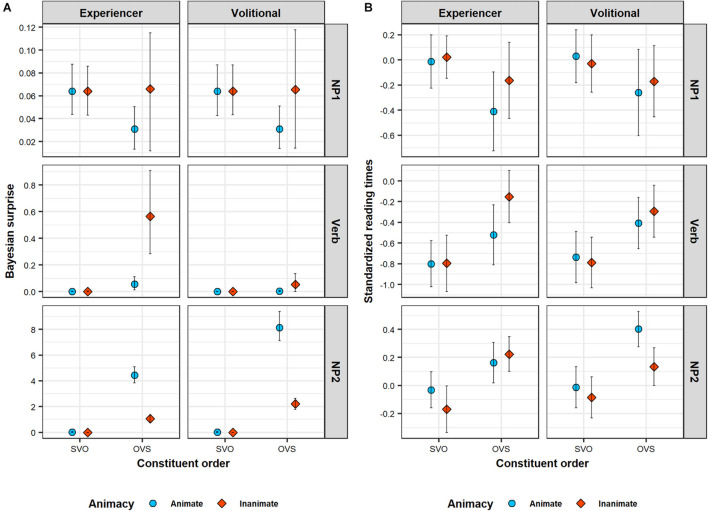
Predicted Bayesian surprise (**Panel A**) compared to length-corrected reading times (**Panel B**) across sentence regions of the critical sentences (rows). Bayesian surprise is derived from the rational model described in Section “A Rational Model of Incremental Argument Interpretation.” Length-corrected reading times are grand averages within each design condition. Note that the range of the y-axis differs between sentence regions. Error bars in **(Panel A)** illustrate 89% across-item average HPDIs of predicted Bayesian surprise, calculated on the basis of the posterior predictions of the underlying GLMMs. Error bars in **(Panel B)** are 89% confidence intervals, calculated on the basis of bootstrapping.

An example item is shown in Table 2. The design fully crosses the constituent order (SVO vs. OVS), verb class (volitional vs. experiencer verb) and the animacy of the direct object (inanimate vs. animate). In the critical sentences, the object is always a lexical NP and therefore lacks case-marking. The subject, on the other hand, is a case-marked pronoun. OVS sentences are therefore morpho-syntactically ambiguous with respect to argument interpretation until the presentation of the post-verbal subject, which disambiguates the sentences toward OVS. In SVO sentences, on the other hand, the pronominal subject is positioned sentence-initially, and morphosyntactic information regarding constituent order is provided directly. The Bayesian surprise of each sentence constituent as predicted by the rational model is illustrated in Panel A of Figure 4. The model predicts that constituent order and object animacy interact in determining Bayesian surprise on NP1: sentence-initial animate nouns lead to less Bayesian surprise than sentence-initial subject pronouns or inanimate nouns. At first, this might seem counter-intuitive, but the effect stems from a stronger bias *in favor* of an SVO interpretation by a subject pronoun than by an animate noun. Whereas the pronoun provides unequivocal support for SVO order, effectively reducing *p*(OS) to zero, the animate noun does not change *p*(OS) as much, keeping it close to the baseline probability of 0.047. An inanimate noun, on the other hand, provides a small effect in the opposite direction, thereby biasing *against* an SVO interpretation. Thus, the rational model predicts somewhat faster RTs for animate nouns in OVS sentences.

**TABLE 2:** Example sentence stimuli of the critical sentences used in the self-paced reading experiment.

Constituent order	Verb	Object animacy	Example
SVO	Volitional	Animate	Jag sparkar killen mitt på smalbenet. ‘I kick the guy in the middle of the shin.’
		Inanimate	Jag sparkar bollen mitt upp i krysset. ‘I kick the ball right up into the top corner.’
	Experiencer	Animate	Jag glömmer killen sent på kvällen. ‘I forget the guy late at night.’
		Inanimate	Jag glömmer bollen mitt på fotbollsplanen. ‘I forget the ball in the middle of the soccer field.’
OVS	Volitional	Animate	Killen sparkar jag mitt på smalbenet. ‘The guy I kick in the middle of the shin.’
		Inanimate	Bollen sparkar jag mitt upp i krysset. ‘The ball I kick right up in the top corner.’
	Experiencer	Animate	Killen glömmer jag sent på kvällen. ‘The guy I forget late at night.’.
		Inanimate	Bollen glömmer jag mitt på fotbollsplanen. ‘The ball I forget in the middle of the soccer field.’

Except in sentences with animate objects and volitional verbs, the Bayesian surprise on the verb is somewhat higher in OVS than in SVO sentences. This difference is particularly pronounced when NP1 is inanimate: the combination of an inanimate NP and either a volitional or experiencer verb provides some additional support for an OVS interpretation, over and above what is provided by the inanimate NP by itself. However, Bayesian surprise is particularly high in OVS sentences with experiencer verbs when NP1 is inanimate in comparison to when it is animate. Here, the combination of an inanimate 3rd-person NP and an experiencer verb work in concert and provide a lot of support for the object-initial interpretation. The rational model thus predicts somewhat slower verb RTs in OVS compared to SVO sentences, particularly in sentences with inanimate objects. Further, it also predicts slower verb RTs in OVS sentences with experiencer verbs when the object is inanimate rather than animate.

At NP2, Bayesian surprise is substantially higher in OVS than in SVO sentences in general, reflecting an increase in the probability of OVS order due to the disambiguating sentence-final subject pronoun (Hörberg et al., 2013). Importantly, however, this increase is strongly mediated by animacy and verb class. Overall, the effect is weaker when the initial object is inanimate. This is because the inanimate NP co-occurring with the verb has already provided some support for the object-initial interpretation, rendering the OVS interpretation more probable. However, the effect of animacy on the probability of OVS order is much more pronounced in sentences with volitional verbs. In experiencer verb sentences, the combination of a 3rd person NP and an experiencer verb has already provided additional support for the OVS interpretation independently of the object’s animacy. The rational model thus predicts slower NP2 RTs in OVS than in SVO sentences. This effect should further be mediated by animacy and verb class in terms of even slower NP2 RTs in OVS sentences with volitional verbs and animate objects.

### Materials and Methods

#### Participants

The self-paced reading experiment was conducted at the Department of Linguistics at Stockholm University. Participants were informed about the experimental procedure and that they could stop at any time without giving reason. They provided written informed consent. A total of 45 participants (15 male) performed the experiment. Their mean age was 28.4 years (*SD* = 9.93), and most of them were students at Stockholm University. Participants received a cinema voucher as reimbursement for their participation.

#### Materials

All sentences consisted of a one-word NP, a single verb, another one-word NP, and a sentence-final prepositional phrase between three to six words long. The stimulus material consists of 32 items, each of which formed an 8-tuple, representing the 2 × 2 × 2 design (as exemplified in Table 2) created from an animate and an inanimate noun, a 1st or 2nd person personal pronoun, and a volitional and an experiencer verb (see Supplementary Table 9 for a full list of these lexical items).

As evident from Table 2, our design implies that a critical item starting with a lexical NP has OVS order. Since there is evidence that readers sometimes learn such experiment-specific statistical contingencies (e.g., Kaschak and Glenberg, 2004; Farmer et al., 2011; Fine et al., 2013; Fraundorf and Jaeger, 2016), we also included three types of SVO filler sentences with lexical subject NPs (see top three rows of Table 3). These filler sentences ensure that sentence-initial nouns occur both as subjects as well as objects, thereby avoiding that sentence-initial nouns become an unambiguous cue to OVS order within the context of the experiment. They consisted of 32 three-tuples of SVO filler sentences in which the lexical objects of the critical sentences instead function as sentence-initial subjects, and post-verbal objects consist of 1st or 2nd person pronouns (with object case-marking). For the animate lexical NPs, we used the same volitional and experiencer verbs as in the critical items. For the inanimate lexical NPs, we had to choose different verbs compatible with inanimate subjects. Additionally, we constructed 32 SVO fillers sentences with 1st and 2nd person pronominal NPs. An example stimulus is shown in the final row of Table 3. A full list of all stimuli is provided in the Supplementary Table 9.

**TABLE 3:** Example sentence stimuli of the filler sentences used in the self-paced reading experiment.

Constituent order	Verb	Subject animacy	Example
SVO	Volitional	Animate	Killen sparkar mig mitt på smalbenet. ‘The guy kicks me in the middle of the shin.’
	Experiencer	Animate	Killen glömmer mig sent på kvällen. ‘The guy forgets me late at night.’
	Inanimate subject verbs	Inanimate	Bollen träffar mig mitt i pannan. ‘The ball hits me in the middle of the forehead.’

All verbs and noun-verb co-occurrences were attested in the 13 billion word Korp collection (Borin et al., 2012). Within each item, different sentence-final prepositional phrases often had to be used in order for the sentences to make sense. Crucially, however, the two initial words of the phrases that directly follow the second NP were held as constant as possible within each item, always consisting of 2–4 letter function words or adverbs that in most cases were identical across sentences within items.

Each experimental sentence was matched with a comprehension question that probed the event described by the corresponding sentence (i.e., *Sparkar han bollen mitt upp i krysset?*—‘Does he kick the ball right up into the top corner?’ for the first example sentence in Table 2). Half of the comprehension questions were correctly answered with a yes, and the other half were to be answered with a no. In some of the “no”-questions the noun, verb, or the sentence-final prepositional phrase of the corresponding experimental sentence was replaced by another noun, verb, or prepositional phrase. In others, the subject and the object of the sentence were exchanged with each other. Each type of “no”-question occurred equally often.

Materials were arranged into four lists, resulting from a repeated Latin square design based on the design. Each participant read one list. First, a repeated Latin-square design was used to distribute the eight critical sentence conditions of each item across four lists so that each list contained two instances of each item. These two instances were chosen such that they did not contain the same nouns or verbs, so that participants did not experience these stimuli as repeated items. This was possible because half of the conditions of each item contained a volitional verb and the other half contained an experiencer verb, and this manipulation was crossed with the animacy of the object. From the perspective of the participant, the two conditions of the items thus appeared unrelated. Across items, we further balanced the number of 1st and 2nd person pronouns in each list.

In order to ensure that participants saw the same sentence-initial nouns in both the subject and object functions, the three SVO filler sentences constructed from each critical item always occurred in a list with a critical OVS sentence from the same item. Within lists, filler sentences with volitional or experiencer verbs always co-occurred with critical sentences with the same verbs. Similarly, filler sentences with inanimate subjects were distributed across lists in a manner that ensured that each inanimate noun both occurred in the subject as well as in the object function. Each list also contained the identical set of 32 SVO filler sentences with 1st and 2nd person pronominal NPs. Each list therefore contained a total of 128 sentences (64 critical sentences, 32 filler sentences varying across lists, and 32 filler sentences that were the same in all lists).

Across participants, each of the four lists were presented in 8 different stimulus orders. Specifically, each list was divided in sequences of eight blocks with 16 sentences each, with item sets, conditions, question types as well as nouns, verbs and pronouns evenly distributed across blocks. Each noun and verb only occurred once within each block. Sentences within a block were presented in a pseudo-randomized fashion so that sentences of the same condition never were presented consecutively. Block order was counterbalanced across participants exposed to each respective list using a Latin square design, ensuring that each block occurred equally often in each of the eight possible list positions. This was done so as to avoid confounding of the conditions of interest with presentation order, since reading times are known to be affected by previous exposure to similar structures (e.g., Fine et al., 2010, 2013; Tooley et al., 2014; Tooley and Traxler, 2018; Yan and Jaeger, 2020).

During data collection, an error in the experimental setup resulted in the first 22 participants being assigned to one of the four lists created from the design factors (order was approximately balanced across those participants). When this error was detected, subsequent participants were exposed to three other lists in a counterbalanced fashion (with 8 participants each, 1 each for each order). Imbalanced data of this type does not violate the assumptions of the analysis approach we employ, and additional statistical analyses not reported here failed to find any significant differences between lists.

#### Procedure

The experiment was performed on a standard personal computer. Before the experimental trials started, written instructions were presented, and participants performed a practice session of 12 practice trials during which they received feedback on their performance.

Each trial consisted of a visual presentation of the sentence using a self-paced moving window paradigm (Just and Carpenter, 1980; Aaronson et al., 1984). First, a fixation cross appeared on the left-hand side of the screen for 800 ms, followed by a 400 ms blank screen. Then, the full sentence was shown with all non-space characters replaced by a hash symbol (#). Participants revealed each consecutive word of the sentence by pressing the space bar with their preferred hand. At each button press, the currently shown word reverted back to hash symbols as the next word was converted to letters, and button press durations were recorded.

After the presentation of the final word, the screen turned blank for 800 ms, and then the comprehension question was shown. The question remained visible until the participant answered it by pressing “y” for ‘yes’ or “n” for ‘no.’ A final blank screen then appeared for 1000 ms before the next trial started. Each experimental block was preceded by a screen that informed that the next block (showing the block number) was about to begin, and the block was started by a space bar press.

#### Data Exclusion and Correction for Word Length

All participants answered the comprehension questions with an accuracy of 80% or higher. Data from all participants was included in the analysis. Following Jegerski (2014), raw RTs below 100 ms or above 4000 ms (0.3% of the data) as well as RTs from incorrectly answered trials (5% of the data) were excluded from further analysis. Following common procedure, RTs were corrected for word length using linear mixed-effects regression: raw RTs were regressed against word length, while controlling for individual variation in RTs and sensitivity to word length across participants, using a by-participants random intercept and slope for word length (e.g., Fine et al., 2013). The residuals of this model are RTs for which the effect of word length and the individual variation and sensitivity to word length has been regressed out. Length-corrected RTs outside of three standard deviations from the participant’s mean were excluded from further analysis (Jegerski, 2014). Taken together, our exclusion criteria removed 7.1% of all RTs from the analysis, leaving 8160 word RTs across the three sentence regions of critical stimuli.^6^

### Results

We present three sets of analyses. We start by assessing the effect of Bayesian surprise on reading times in each of the three sentence regions (NP1, verb, NP1). This analysis tests whether the prediction error caused by changes in expectations—under a Bayesian surprise linking hypothesis—predicts variation in reading times. For comparison to previous work, our second set of analyses assesses the effect of linguistic cues—constituent order (OVS vs. SVO), animacy (inanimate vs. animate), verb class (volitional vs. experiencer), and their interactions—on reading times. These analyses parallel previous work that has investigated effects of linguistic cues on sentence processing (e.g., Ferreira and Clifton, 1986; Trueswell et al., 1994; Gennari and MacDonald, 2008; Wu et al., 2010). This second set of analyses also allows us to assess whether the effects of linguistic cues *qualitatively* follow the prediction of the rational model (whereas our first set of analysis focus on the quantitative fit). Third, we ask whether the effects of linguistic cues on reading times are fully accounted for by Bayesian surprise—the prediction error resulting from *expectations* based on those cues. Additional analyses reported in the Supplementary Section 7, show that the effects of Bayesian surprise can*not* be reduced to word-level surprisal—a measure that can be seen as approximating the Bayesian surprise across *all* levels of linguistic processing (Levy, 2008), and that has been found to be a good predictor of reading times (e.g., Frank and Bod, 2011; Smith and Levy, 2013; Brothers and Kuperberg, 2021).

All analyses employed Bayesian mixed-effects linear regression (LMM), again using the package *brms* (Bürkner, 2017, 2018) in *R* (R Core Team, 2020). The use of Bayesian, rather than frequentist, data analysis facilitates convergence under the full random effect structure (for an overview of additional advantages, see Wagenmakers, 2007). We used the standard weakly regularizing priors as recommended in the literature (e.g., Gelman, 2006; Gelman et al., 2008; Stan Development Team, 2017). For fixed effect parameters, we use 3 degree of freedom Student *t* priors with a mean of zero and a standard deviation of 2.5 units (following Gelman et al., 2008). For random effect standard deviations, we use a Cauchy prior with location 0 and scale 2. For random effect correlations, we use an LKJ-Correlation prior with the shape parameter set to 1 (Lewandowski et al., 2009), describing a uniform prior over correlation matrices. All analyses were fit using 12 chains with 1,000 warmup-samples and 4000 post-warmup samples per chain, resulting in 48,000 posterior samples for each analysis. In the Supplementary Section 6, we report frequentist analyses paralleling those presented here.

#### Effects of Bayesian Surprise

In order to evaluate the quantitative relationships between RTs and Bayesian surprise, we conducted separate LMMs for the NP1, verb, and NP2 regions, marked in example (3). Whereas NP1 and verb RTs were RTs of individual words (i.e., the initial single-word NP and the verb), NP2 RTs consisted of the region-averaged RT of the one-word, post-verbal NP and the initial word of the upcoming adverbial. This decision was made prior to data analysis, following the common approach to spill-over effects to capture effects that affect button presses on immediately subsequent words (Mitchell, 1984, among many others). All analyses reported in the main text are based on length-corrected RTs that averaged over the sentence regions exemplified in example (3). For the sake of comparison, the result figures we present below also show region-averaged RTs for the subsequent “adverbial region”, consisting of the subsequent two words of the adverbial, as well as RTs of the sentence-final word.


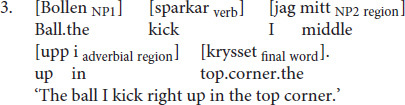


We used standardized Bayesian surprise as the only fixed-effect predictor in the LMMs. Only by-participant intercepts were included since more complex random effect structures did not converge.^7^ Model summaries contain maximum a posteriori (MAP) parameter estimates, corresponding 89% highest posterior density intervals (HPDIs), and the posterior probability (*p*_*posterior*_) of the parameter taking on values in the direction of the MAP parameter estimate. These were obtained with the *describe_posterior()* function in R package *BayestestR* (Makowski et al., 2019).

**FIGURE 5:**
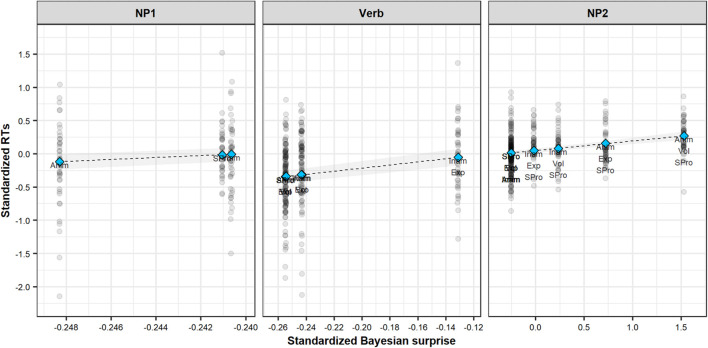
Bayesian LMM MAP estimates of standardized RTs in each sentence region as function of standardized Bayesian surprise. Shaded areas illustrate 89% HPDIs. Shaded dots represent individual item-level RTs. Texts describe item-level sentence properties. Anim: animate; Inam: inanimate; Pro: pronominal; S: subject; O: object; Vol: volitional; Exp: experiencer.

We find very clear evidence for a positive effect of Bayesian surprise for all three sentence regions (NP1: ${\hat{\beta }}_{MAP}$ = 14.41, SE = 6.21, HDPI = [3.53, 23.50], *p*_*posterior*_ = 0.996; Verb: ${\hat{\beta }}_{MAP}$ = 2.35, SE = 0.48, HDPI = [1.58, 3.12], *p*_*posterior*_ = 1.000; NP2: ${\hat{\beta }}_{MAP}$ = 0.143, SE = 0.03, HDPI = [0.10; 0.19], *p*_*posterior*_ = 1.000). These relationships are illustrated in Figure 5.

#### Effects of Linguistic Cues

Next, we analyzed the qualitative effects of linguistic cues (animacy, verb semantics, and constituent order) on the same three sentence regions. This facilitates the comparison to previous work, and sheds further light on the qualitative relation between the reading time patterns associated with linguistics cues and the predictions of the rational model.

The LMM of the NP1 region contained fixed effects for object animacy (sum-coded: 0.5 = animate vs. −0.5 = inanimate), constituent order (sum-coded: 0.5 = SVO vs. −0.5 = OVS), and the animacy × order interaction. The LMMs of the verb and NP2 region contained fixed effects for object animacy (same coding as for NP1), constituent order (same coding as for NP1), and verb (sum-coded: 0.5 = experiencer vs. −0.5 = volitional), as well as the full factorial interactions.^8^ All LMMs also included the maximal random effect structure by-participants—i.e., by-participant random intercepts and slopes for all predictors in the analysis. No by-item random effects were included, since inclusion led to failure to converge (see text footnote 7).

**FIGURE 6:**
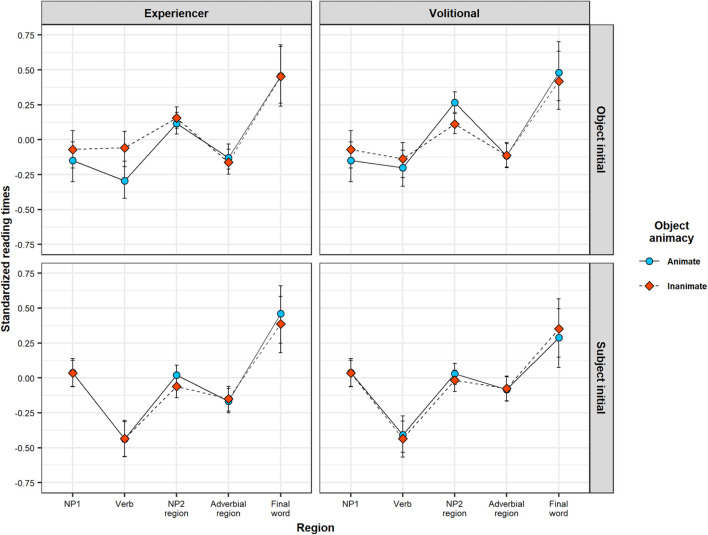
Bayesian LMM model MAP estimates of standardized RTs in each sentence region as function of animacy, separated by verb class (experiencer vs. volitional) and constituent order (OVS vs. SVO). Error bars illustrate 89% HPDIs.

The results are summarized in Table 4. Figure 6 illustrates predicted RTs across sentence regions, as a function of linguistic cues.

**TABLE 4:**
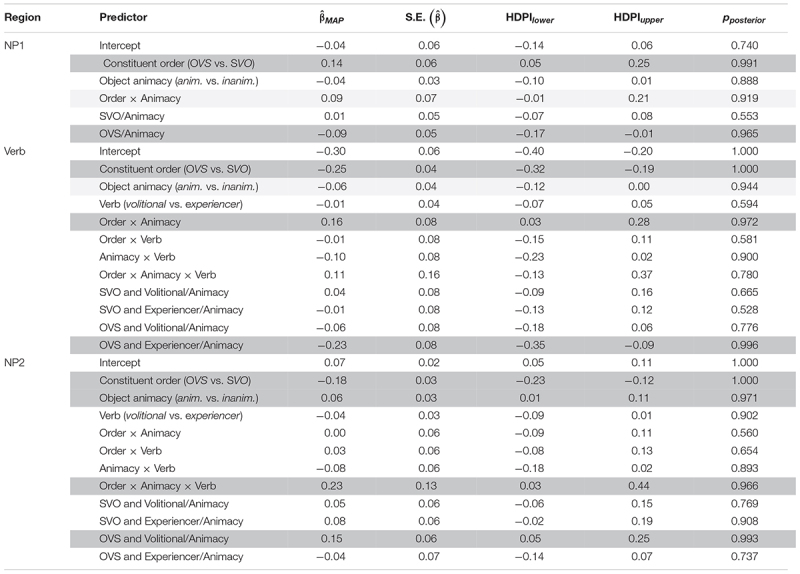
Results of the Bayesian linear mixed-effects regressions (LMMs) of region-averaged length-corrected RTs investigating the effects of linguistic cues over the NP1, verb, and NP2 region.

*For each region, we show both the main LMM (top) and simple effects re-parameterization of the same LMM. The first number column provides the maximum a posteriori probability estimates for each coefficient $\left({\hat{\beta }}_{MAP}\right)$, the standard error of that estimate, the lower and upper bounds of the 89% highest posterior density interval (HPDI, following Kruschke, 2014), and the posterior probability that the effect has the sign of the MAP estimate. Effects that meet conventional frequentist significance criteria are highlighted by shading (p_posterior_ > 0.95).*

For the NP1 region, we found a main effect of constituent order: length-corrected RTs were slower in SVO sentences (where NP1 is a case-marked pronoun) than in OVS sentences (where NP1 is a lexical noun). There was also evidence for an interaction between constituent order and object animacy, although this evidence did not reach the conventional frequentist threshold of significance. Simple effect analyses (see Table 4) showed that the effect of constituent order is primarily driven by the shorter RTs for animate object nouns in OVS sentences.

Of note is that the linguistic LMMs could—in theory—accommodate effects of animacy and constituent order in any direction and of any magnitude. Yet, this analysis finds that RTs on NP1 pattern in ways that closely resemble the qualitative predictions derived from the rational model of argument interpretation presented in Section “Testing the Predictions of the Rational Model Against Human Reading Times.” Figure 4 provides a direct comparison between patterns of predicted Bayesian surprise (Panel A) and average RTs (Panel B). In line with the predictions of the rational model, RTs are shorter for animate NP1s on OVS sentences, compared to all other conditions. Notably, these lexical NP1s in OVS sentences were read faster even than subject pronouns NP1s (in SVO sentences). This is the case despite the fact that subject pronouns are case-marked and thus morphologically unambiguous with respect to argument interpretation. Under the rational model, this makes sense: sentence-initial animate NPs do not provide much support in favor of either argument interpretation, leading to low Bayesian surprise. A subject pronoun, on the other hand, provides unequivocal support for an SVO interpretation. This support goes against the small but nevertheless existing expectation for OVS order, leading to comparatively larger Bayesian surprise (Similarly, an *in*animate lexical NP1 provides some additional support in favor of an OVS interpretation, violating the overall baseline expectation for SVO order, also leading to higher Bayesian surprise than the animate lexical NP1).

For the verb region, we again found a main effect of constituent order, but in the opposite direction than for the NP1 region: RTs were slower in OVS sentences (where the verb follows a non-case marked lexical noun) than in SVO sentences (where the verb follows a case-marked subject pronoun). In addition, evidence for an interaction of this effect with animacy reached the conventional frequentist threshold of significance. Simple effect analyses (see Table 4) found that object animacy affected verb RTs primarily for sentences with OVS order: verb RTs in OVS sentences were slower when the verb was preceded by an inanimate object noun than when it was preceded by an animate object noun. Simple effects analyses further showed that this effect of object animacy on verb RTs in OVS sentences was particularly pronounced for experiencer verbs.

For the verb region, too, the linguistic LMM thus returns effects that follow the qualitative predictions of the rational model (see Figure 4). The combination of an inanimate NP1 and either a volitional or experiencer verb provides some support for an OVS interpretation, over and above what is provided by the inanimate NP by itself. In contrast to what is observed for NP1 RTs, the rational model thus predicts verb RTs to be slower in OVS with inanimate objects. Further, because experiencer verbs frequently occur with 1st or 2nd person subjects (Dahl, 2000), the co-occurrence of a 3rd-person initial NP and an experiencer verb provides additional support for OVS order. Verb RTs are therefore predicted to be particularly slow in OVS sentences with an experiencer verb and an inanimate NP1.

Finally, for the NP2 region, we found a main effect of constituent order in the same direction as on the verb: NP2 RTs were slower in OVS sentences (where NP2 is a subject pronoun) than in SVO sentences (where NP2 consists of an object noun). There was also a main effect of animacy, showing that NP2 RTs overall are slower when the object noun is animate, irrespective of the position of the object. These effects need to be interpreted in light of the three-way interaction between constituent order, object animacy, and verb class. Simple effect analyses (see Table 4) found that NP2 RTs in OVS sentences are slowed down when the sentence-initial noun is animate—but only in sentences with volitional verbs. In OVS sentences with experiencer verbs, this animacy-induced slow-down instead already occurred on the verb.

Again, this RT pattern is qualitatively in line with the predictions of the rational model of argument interpretation (see Figure 4), and can be explained in terms of changes in the expectation for OVS word order. The sentence-final subject pronoun in OVS sentences disambiguates the sentence interpretation toward OVS. The slowdown on the NP2 for OVS sentences in comparison to SVO sentences is a predicted consequence of this change in expectations. The magnitude of this change depends on the extent to which NP1 animacy and verb class provides support for an OVS interpretation before NP2 has been encountered. In particular, an animate NP1 combined with a volitional verb provides no additional support for OVS word order prior to the presentation of NP2. The sentence-final subject pronoun is therefore highly unexpected in such sentences, resulting in particularly slow RTs.

#### Can Bayesian Surprise Capture the Effects of Linguistic Cues on Reading Times?

In order to evaluate how much of the effects of linguistic cues Bayesian surprise can account for, we performed separate model comparisons for each of the three sentence regions. For each region, we refit the separate analyses of (i) Bayesian surprise and (ii) linguistic cues presented above but while including the full random effect structure from *both* analyses. Following recommendation for model comparison, the linguistic LMM and the Bayesian surprise LMM thus only differ in terms of their fixed effects.

We compare LMMs in terms of their out-of-sample predictive accuracy—the LMM’s leave-one-out cross-validation information criterion (LOOIC—see Watanabe, 2013; Gelman et al., 2014; Vehtari et al., 2017). This LOOIC is related to an LMM’s leave-one-out cross-validated log predictive density or *elpd*_*LOO*_ (LOOIC = −2 × elpd_*LO*__*O*_) in the same way that an LMM’s deviance is related to its log-likelihood (deviance = −2 × log-likelihood). Smaller LOOICs indicate better predictive accuracy, similar to traditional deviance measures of model fit (e.g., the AIC or BIC). Unlike measures based on the log-likelihood, the elpd measures how well the LMM generalizes to held-out data. This takes into account the models’ functional flexibility (which can lead to good fit on the observed sample but poor generalization to novel data). Additional analyses presented in the Supplementary Table 11, report model comparisons based on likelihood ratios, which captures the model’s fit against the finite sample the researcher analyses. Unlike model comparison based on likelihood ratios, the elpd is not limited to comparison of nested models. This allows us to directly compare the linguistic and Bayesian surprise LMMs without comparing them indirectly through pairwise comparison to a superset LMM with the predictors from both LMMs.

The goal of the model comparison we conduct here is to assess to what extent reading time predictions based on linguistic cues are accounted for by Bayesian surprise with a single degree of freedom (DF). This conclusion would be supported if the Bayesian surprise LMM outperforms the linguistic LMM, or if the Bayesian surprise and linguistic LMMs do *not* differ in terms of their elpd. The latter outcome would indicate that the two LMMs achieve the same predictive accuracy but the Bayesian surprise LMM would do so with fewer DFs: each Bayesian surprise LMM only has a single DF in predicting RTs (all other DFs are fixed based on the corpus data, as described in Section “A Rational Model of Incremental Argument Interpretation”); the linguistic LMMs, however, have up to 7 DFs (resulting from the 2 × 2 × 2 design). If, however, the linguistic LMM outperforms the Bayesian surprise LMM, this would argue that the linguistic model—with its additional flexibility—can capture important predictive information about reading times that are not captured by the rational model that links changes in expectations to reading times.

We report differences in the LOOIC (ΔLOOIC). Following Bushong (2020), we consider a difference in LOOIC of more than 2.5 times of its estimated standard error (i.e., estimated differences outside the 99% error interval of the difference) as evidence for a difference in predictive accuracy between the models. Table 5 summarizes the results. The Bayesian surprise LMM has a numerically better LOOIC than the linguistic LMM for both the NP1 and NP2 region, and vice versa for the verb region. However, all of these numerical differences fall well within the 99% interval. We thus do not have evidence that the two LMMs differ in their predictive accuracy at any of the three sentence regions. This suggests that Bayesian surprise largely captures the same predictive information about RTs as a model including the individual linguistic cues.

**TABLE 5:** Out-of-sample predictive accuracy of linguistic and Bayesian surprise LMMs for each sentence region.

Constituent	LOOIC		LOOIC differences (ΔLOOIC)			
	Linguistic	Bayesian surprise	Estimate	S.E.	Lower	Upper
NP1	6748.29	6746.13	2.16	2.98	−5.3	9.61
Verb	7725.25	7735.99	10.74	8.01	−9.29	30.76
NP2	6665.08	6664.97	0.12	5.92	−14.68	14.92

*We compare models in terms of leave-one-out cross-validated (LOO) log predictive density (elpd_LOO_), specifically the difference ΔLOOIC between LMMs in the LOO information criterion (LOOIC = −2 × elpd_LOO_). Confidence intervals for the differences are 2.5 standard errors below and above each difference. A confidence interval excluding zero is considered as evidence for a difference in predictive accuracy between the models at hand.*

## General Discussion

Previous research has shown that the incremental interpretation of arguments is based on an interplay between form-based morpho-syntactic, meaning-based semantic and discourse-pragmatic NP properties, and verb-semantic cues (e.g., MacWhinney and Bates, 1989; MacDonald et al., 1994; Bornkessel and Schlesewsky, 2006; Bornkessel-Schlesewsky and Schlesewsky, 2009). On *linguistic accounts*, some of these cues—e.g., the prominence properties of arguments—are assumed to have a privileged role in language comprehension (Bornkessel and Schlesewsky, 2006; Kuperberg, 2007; Alday et al., 2014; see also Nakano et al., 2010; Szewczyk and Schriefers, 2011). For example, these cues might be assumed to be processed first, prior to other cues (Bornkessel and Schlesewsky, 2006), or to be processed by a separate mechanism (Kuperberg, 2007: 37).

In contrast, *linguistic accounts* attribute the effects of linguistic cues to implicit expectations based on the joint distribution of cues and argument assignments in previously experienced language input (e.g., MacDonald et al., 1994; Trueswell et al., 1994; McRae et al., 1998; Narayanan and Jurafsky, 1998; Kempe and MacWhinney, 1999; Vosse and Kempen, 2000, 2009; Tily, 2010; MacDonald, 2013; Bornkessel-Schlesewsky and Schlesewsky, 2019; Rabovsky, 2020).

The present study compared linguistic accounts of incremental argument interpretation, in which cues to argument interpretation have a direct effect, to expectation-based accounts, in which the cues are mediated through expectations. To this end, we developed a rational expectation-based model of incremental argument interpretation in simple transitive clauses in Swedish and then tested this model against reading time data from a self-paced reading experiment. The rational model predicts processing costs at different sentence regions for different constituent orders, morpho-syntactic and prominence properties of the NP arguments, and semantic properties of the verb. It estimates the incremental change in expectations about argument interpretation as a function of the cues provided by the subsequent sentence constituents (i.e., NP1, verb, and NP2), quantified in terms of Bayesian surprise—a shift from a prior to a posterior probability for a particular argument assignment.

We tested some of the most prominent predictions of this rational model against processing times in a moving window self-paced reading experiment of transitive sentences in Swedish. The model predicts that the processing difficulty associated with argument interpretation in locally ambiguous sentences depends on an interplay between prominence properties of the initial NP and the semantic class of the verb (see Figure 3). In particular, processing difficulty is predicted to vary as a function of the animacy of NP1 and whether the sentence verb is volitional or experiencer. We therefore used locally ambiguous OVS sentences and unambiguous SVO sentences with lexical objects and case-marked subject pronouns that varied with respect to the animacy of the object and whether the verb was volitional or experiencer. The results of the experiment confirmed most of the predictions of the rational model both quantitatively—Bayesian surprise is a significant predictor of within-region RTs (Figure 5)—and qualitatively—the effects of linguistic cues on RTs pattern similarly to their effect on Bayesian surprise (Figure 4). In all regions, higher Bayesian surprise predicted higher reading times, and the observed patterns of effects could be explained in terms of changes in the expectation for OVS order (see Section “Effects of Linguistic Cues”). This pattern of results is predicted under the hypothesis that listeners incrementally update their expectations about argument interpretations, with larger changes in expectations requiring more processing time.

In order to more directly compare the linguistic account of argument interpretation to the expectation-based account, we further investigated whether Bayesian surprise can predict RTs just as well as a model in which linguistic cues can have arbitrary direct effects on RTs. We found no evidence that direct effects of linguistic cues (as predicted by the linguistic account of argument interpretation) are required to predict RTs beyond the effects mediated through Bayesian surprise (as predicted by the rational expectation-based account). Thus, with only a single degree of freedom, Bayesian surprise derived from our rational model seems to achieve predictive accuracy for reading times that is about equally high as for the functionally much more flexible linguistic account.

At first blush, this finding might be surprising given that some previous studies have concluded that frequency information is insufficient to explain the interactions between different linguistics cues (Mitchell, 1987; Gibson et al., 1996; Pickering et al., 2000; Kennison, 2001; Van Gompel and Pickering, 2001; Bornkessel et al., 2002; McKoon and Ratcliff, 2003). For example, Bornkessel et al. (2002) compared ERP responses associated with initial nominative-, accusative-, or dative-marked NPs in German complement clauses. In this sentence context, both accusative- and dative-marked NPs are infrequent, compared to nominate-marked NPs. Frequency-based accounts of argument interpretation, Bornkessel and colleagues argued, would thus predict increased processing costs—and hence enhanced amplitude of the N400 response—for both accusative and dative NPs, compared to nominate NPs. In contrast to this prediction, Bornkessel and colleagues observed increased N400 amplitudes only for accusative NPs. Critically though, this does not rule out expectation-based accounts of argument interpretation. As we have summarized here (but see also earlier works, e.g., McRae et al., 1998), the relevant theoretical construct in expectation-based accounts are the *contextual* expectations. These are based on the *conditional* probability distribution of argument assignments given the available cues (incl. the properties of the initial NP and the preceding context), not the overall frequency of different argument assignments. An interesting question for future work is thus to see whether results like those of Bornkessel et al. (2002) could be accounted for by a model like the one we presented here.

Taken together, these findings argue against accounts that attribute a privileged role to some types of cues (Bornkessel and Schlesewsky, 2006; Kuperberg, 2007; Alday et al., 2014; see also Nakano et al., 2010; Szewczyk and Schriefers, 2011). Instead, our findings provide further support for expectation-based accounts of incremental argument interpretation: the effects of morpho-syntactic, argument prominence and verb-semantic cues on argument interpretation seem to be indirect, mediated through implicit expectations that are based on the distribution of these cues in previous language input. Our results thus corroborate findings from earlier work on probabilistic sentence comprehension (MacDonald et al., 1994; Garnsey et al., 1997; Tabor et al., 1997; McRae et al., 1998; Spivey-Knowlton and Tanenhaus, 1998; Vosse and Kempen, 2000, 2009, among many others). In competition-based models, for example, the processing difficulty of argument interpretation is determined by the extent to which the cues introduced at the current sentence region disagree with the relative activation of competing argument assignments at the preceding sentence region. The present approach borrows from, and builds on, these previous works (see Levy, 2008 for a nuanced discussion of commonalities and differences between rational and competition accounts). Unlike earlier accounts, however, the rational model presented here does not contain any hidden parameters, thereby putting the expectation-based hypothesis to a stronger test. As far as we know, the present work is the first to directly pit the expectation-based account against a linguistic account, by directly comparing the rational model to a linguistic model with respect to their out-of-sample predictive accuracy.

A long line of research has entertained the idea that language comprehension is expectation-based and draws on statistical patterns in the input (for reviews, see MacDonald, 2013; Kuperberg and Jaeger, 2016). However, most of this work—in particular within the rational tradition—has focused on expectations for individual words, parts-of-speech, or syntactic parses (e.g., Hale, 2001; Demberg and Keller, 2008; Levy, 2008, 2011; Smith and Levy, 2013; Linzen and Jaeger, 2014; Frank et al., 2015; Brothers and Kuperberg, 2021). The present study is instead concerned with argument interpretation—the process by which the NP arguments are “assigned” or “linked” to the argument-slots required by the verb. Unlike models of word-level surprisal, the rational model introduced here transparently links linguistic cues to their effect on the probability of argument assignments. This, we hope, will facilitate transfer from, and comparison to, linguistic accounts, which have typically focused on the role of specific cues. For example, the rational model of argument interpretation allows us to quantify and predict the *magnitude of effects* associated with different types of linguistic cues (Figure 3 above as well as Supplementary Figures 4, 5 and Supplementary Tables 6–8). This makes apparent which cues are particularly important to argument interpretation, and how different cues interact. Additional analyses presented in the Supplementary Section 7, further found that Bayesian surprise over argument assignment captures different aspects of reading times than a model of word-level surprisal. This suggests that expectation-based models of argument interpretation might bridge the gap between expectation-based accounts of word-level surprisal and linguistic accounts of argument interpretation.

An obvious limitation of our *model*—as opposed to the general proposal to estimate Bayesian surprise over argument assignments—is that it only applies to Swedish transitive sentences presented in isolation. It thus implicitly assumes that the comprehender knows—or strongly expects— that all sentences have a subject and an object whose relative ordering is to be determined, and that the baseline probability of the two competing orders are always the same. Although these assumptions are likely to be warranted in the context of our experiment—where unrelated transitive sentences are presented in isolation— it is clearly violated for argument interpretation in natural discourse contexts. Although there is more uncertainty about, for instance, the number and types of NP arguments in sentences in natural language, there is also additional information about NP argument functions, as word order variations primarily are motivated by discourse-pragmatic relations (such as topic and contrast, see Hörberg, 2016, 2018). The theoretical proposal made here predicts that such discourse-pragmatic information plays an important role in argument interpretation in the processing of natural language, although the simple model we test here would not be able to account for them.

With that being said, the rational model tested here makes predictions for a wide variety of transitive clauses with different syntactic configurations (i.e., NP- versus adverbial-initial, with or without auxiliary verbs, with or without sentential adverbials, and with NP arguments of any length), and draws upon many different properties (nine NP properties, four verb-semantic classes, and two syntactic properties; see Table 1). The present experiment tested only a small subset of the predictions even this simple model makes. Future work could thus use the same model to derive predictions for further experiments, contrasting other sentence types and/or other linguistic cues that the model includes. Other experimental paradigms and/or more high-powered experimental designs should be able to detect more subtle effects that the model predicts.

## Summary

Incremental argument interpretation draws on an interplay between form-, meaning- and discourse-based argument properties, and verb-semantic information, that function as cues to argument assignment during incremental sentence comprehension. We have provided evidence for the hypothesis that the effects of these cues to argument interpretation are mediated through expectations, based on their joint distribution over NP arguments in previously experienced language input. Based on the distribution of these cues in a corpus of transitive sentences in Swedish, we develop a rational model of incremental argument interpretation. This model predicts the processing difficulty experienced at each sentence constituent (i.e., NP1, verb, and NP2) as a function of the Bayesian surprise associated with changes in expectations over possible argument interpretations. The predictions of the rational model were found confirmed by reading times from a self-paced reading experiment of Swedish transitive sentences, both quantitatively, by directly predicting reading times, and qualitatively, in terms of showing similar patterns with respect to linguistic cues.

## Data Availability Statement

The datasets presented in this study can be found in the Open Science Framework (OSF) repository: https://osf.io/rw5nf/.

## Ethics Statement

Ethical review and approval was not required for the study on human participants in accordance with the local legislation and institutional requirements. The participants provided their written informed consent to participate in this study.

## Author Contributions

Both authors wrote the manuscript and conceived of the general ideas underlying the rational model, with TFJ suggesting the theoretical framework behind it. With input from TFJ, TH collected the corpus data, designed the implementation of the rational model, designed and performed the self-paced reading experiment, and performed the statistical analyses.

## Author Disclaimer

The views expressed here are not necessarily those of the funding agencies.

## Conflict of Interest

The authors declare that the research was conducted in the absence of any commercial or financial relationships that could be construed as a potential conflict of interest.

## Publisher’s Note

All claims expressed in this article are solely those of the authors and do not necessarily represent those of their affiliated organizations, or those of the publisher, the editors and the reviewers. Any product that may be evaluated in this article, or claim that may be made by its manufacturer, is not guaranteed or endorsed by the publisher.

## Footnotes

1 We use the terms argument interpretation and argument role assignment to refer to the process of “assigning” or “linking” arguments to the argument-slots required by the verb (Van Valin, 2006). For example, the transitive verb “kick” requires an actor and an undergoer of the kicking action.

2 Throughout this paper, we use the assignment of *grammatical functions*—specifically, subjects and (direct) objects—to operationalize the assignment of argument roles—specifically, actor and undergoer roles. The two processes are not the same, and it is unclear whether sentence understanding *requires* grammatical function assignment, depending on the grammatical system of the language (Van Valin and LaPolla, 1997; Van Valin, 2005; for review, see Bickel, 2010) or even the “depth of processing” (e.g., Ferreira, 2003). However, for the type of clauses we test the rational model against here (Swedish transitive clauses in active voice), grammatical function determines argument assignment (as defined here, and in more detail in Hörberg, 2016: 8–10). If one was to scale the rational model we present here beyond the scope of the present study, it is important to keep in mind the distinction between argument roles and grammatical functions.

3 For Swedish transitive clauses, for example, Dahl (2000) found that 93.2% of the subjects and 9.9% of the objects were animate, and that 60.7% of the subjects but only 2% of the objects were 1st or 2nd person pronouns.

4 During natural reading, the information available at constituent *C*_*i*_ might include information available through parafoveal preview from upcoming constituents. Under a rational account of reading, this information is expected be weighted less strongly as parafoveal preview has less visual resolution, resulting in increased uncertainty about the input (for related discussion, see Bicknell and Levy, 2012; Kliegl et al., 2013; Bernard and Castet, 2019). In the self-paced reading experiment we present below, this possibility is severely limited since only information regarding the *length* of neighboring words is available parafoveally in a moving-window display.

5 One exception is that we varied the grammatical number (singular vs. plural) of NP1 and NP2 *between* items. This decision was made in order to avoid that all stimuli have identical structure. As confirmed in Figure 3, the effect of number on Bayesian surprise—and thus its predicted effect on RTs—is very small. We thus do not discuss it further. The negligible effect of number is also the reason why we do not take these cues into account in the *linguistic* model below, since inclusion of number (or additional predictors that do not vary across items) in the linguistic model would unfairly bias the model comparison *against* the linguistic account (making the linguistic model more complex without commensurate improvements in expected fit). Additionally, our design varied the person of the subject pronoun (1st vs. 2nd) between items. This, however, does not have *any* effect on the predictions of the model since our rational model only contrasts speech act participants (1st and 2nd person) against non-speech act participants (3rd person), as it is this difference that primarily differentiates subjects and objects (e.g., Dahl, 2000).

6 Additional analyses requested by a reviewer used a different approach to word length correction. Following the reviewer’s suggestion, we analyzed log-transformed RTs, instead of length-corrected RTs, and included word length as an additional predictor in the main analysis rather than first regressing it out of the RTs. These alternative analyses largely yield the same results as reported here, except that neither Bayesian surprise, nor linguistic cues any longer had significant effects on NP1 RTs. Since these alternative analyses also did not address the convergence issues described in text footnote 7, we do not present them in further detail.

7 By-item random intercepts or slopes likely did not converge because lexical content varied as much *within* items (see Table 2) as it did across items. There is thus little systematic cross-item variance (see Supplementary Figure 6). Additionally, Bayesian surprise varied almost exclusively by condition and thus *within* but not across items: by design, 99.97% (NP1 region), 99.99% (verb), and 99.99% (NP2) of the total variance in Bayesian surprise was accounted for by the eight design conditions. The remaining 0.01–0.03% variance is due to the fact that items differed in whether they employed singular or plural lexical NPs. This is a property that the rational model predicts to have small effect on Bayesian surprise and thus on RTs (see Figure 3).

8 The inclusion of SVO vs. OVS order, which is *not* a linguistic cue but rather the variable to be inferred during argument interpretation, might seem counterintuitive. Here constituent order and its interaction with animacy together encode what cues occur in the three sentence regions of our stimuli. The predictor we refer to as constituent order encodes whether the NP1 is a case-marked pronoun or a non-case marked lexical noun.

## References

[B1] Aaronson D. Ferres S. Kieras D. E. Just M. A. (1984). “The Word-by-Word Reading paradigm: An Experimental and Theoretical Approach,” in *New Methods in Reading Comprehension Research*, eds Kieras D. E. Just M. A. (London: Lawrence Erlbaum Associates, Inc), 31–68. 10.4324/9780429505379-3

[B2] Acuña-Fariña C. Fraga I. García-Orza J. Piñeiro A. (2009). Animacy in the adjunction of Spanish RCs to complex NPs. *Eur. J. Cogn. Psychol.* 21 1137–1165. 10.1080/09541440802622824

[B3] Alday P. M. Schlesewsky M. Bornkessel-Schlesewsky I. (2014). Towards a Computational Model of Actor-Based Language Comprehension. *Neuroinformatics* 12 143–179. 10.1007/s12021-013-9198-x 23912508

[B4] Bernard J.-B. Castet E. (2019). The optimal use of non-optimal letter information in foveal and parafoveal word recognition. *Vision Res.* 155 44–61. 10.1016/j.visres.2018.12.006 30629974

[B5] Bickel B. (2010). “Grammatical Relations Typology,” in *The Oxford Handbook of Linguistic Typology*, ed. Song J. J. (Oxford: Oxford University Press), 399–444. 10.1093/oxfordhb/9780199281251.013.0020

[B6] Bicknell K. Levy R. (2012). “Why long words take longer to read: the role of uncertainty about word length,” in *Proceedings of the 3rd Workshop on Cognitive Modeling and Computational Linguistics (CMCL 2012)*, (Montréal: Association for Computational Linguistics), 21–30.

[B7] Bock K. J. Irwin D. E. (1980). Syntactic effects of information availability in sentence production. *J. Verbal Learn. Verbal Behav.* 19 467–484. 10.1016/S0022-5371(80)90321-7

[B8] Bock K. J. Warren R. K. (1985). Conceptual accessibility and syntactic structure in sentence formation. *Cognition* 21 47–67. 10.1016/0010-0277(85)90023-X 4075761

[B9] Borin L. Forsberg M. Roxendal J. (2012). “Korp - the corpus infrastructure of Språkbanken,” in *Proceedings of LREC 2012*, (Istanbul: LREC), 474–478.

[B10] Bornkessel I. Schlesewsky M. (2006). The extended argument dependency model: A neurocognitive approach to sentence comprehension across languages. *Psychol. Rev.* 113 787–821. 10.1037/0033-295X.113.4.787 17014303

[B11] Bornkessel I. Schlesewsky M. Friederici A. D. (2002). Grammar overrides frequency: Evidence from the online processing of flexible word order. *Cognition* 85 B21–B30. 10.1016/S0010-0277(02)00076-8 12127701

[B12] Bornkessel-Schlesewsky I. Schlesewsky M. (2009). Minimality as vacuous distinctness: Evidence from cross-linguistic sentence comprehension. *Lingua* 119 1541–1559. 10.1016/j.lingua.2008.03.005

[B13] Bornkessel-Schlesewsky I. Schlesewsky M. (2019). Toward a Neurobiologically Plausible Model of Language-Related, Negative Event-Related Potentials. *Front. Psychol.* 10:298. 10.3389/fpsyg.2019.00298 30846950 PMC6393377

[B14] Bornkessel-Schlesewsky I. Kretzschmar F. Tune S. Wang L. Genç S. Philipp M. (2011). Think globally: Cross-linguistic variation in electrophysiological activity during sentence comprehension. *Brain Lang.* 117 133–152. 10.1016/j.bandl.2010.09.010 20970843

[B15] Boston M. F. Hale J. T. Vasishth S. Kliegl R. (2011). Parallel processing and sentence comprehension difficulty. *Lang. Cogn. Process.* 26 301–349. 10.1080/01690965.2010.492228

[B16] Bouma G. J. (2008). *Starting a Sentence in Dutch.* Ph. D. thesis. Groningen: University of Groningen.

[B17] Brothers T. Kuperberg G. R. (2021). Word predictability effects are linear, not logarithmic: Implications for probabilistic models of sentence comprehension. *J. Mem. Lang.* 116:104174. 10.1016/j.jml.2020.104174 33100508 PMC7584137

[B18] Bürkner P.-C. (2017). brms: An R Package for Bayesian Multilevel Models Using Stan. *J. Stat. Softw.* 80 1–28. 10.18637/jss.v080.i01

[B19] Bürkner P.-C. (2018). Advanced Bayesian Multilevel Modeling with the R Package brms. *R J.* 10 395–411. 10.32614/RJ-2018-017

[B20] Bushong W. (2020). *Maintenance of Subcategorical Information in Spoken Word Recognition.* Ph. D. thesis. Rochester: University of Rochester.

[B21] Crocker M. W. Brants T. (2000). Wide-Coverage Probabilistic Sentence Processing. *J. Psycholinguist. Res.* 29 647–669. 10.1023/A:1026560822390 11196067

[B22] Czypionka A. Spalek K. Wartenburger I. Krifka M. (2017). On the interplay of object animacy and verb type during sentence comprehension in German: ERP evidence from the processing of transitive dative and accusative constructions. *Linguistics* 55:0031. 10.1515/ling-2017-0031

[B23] Dahl Ö (2000). Egophoricity in discourse and syntax. *Funct. Lang.* 7 37–77. 10.1075/fol.7.1.03dah 33486653

[B24] Dahl Ö Fraurud K. (1996). “Animacy in grammar and discourse,” in *Reference and referent accessibility, Pragmatics & Beyond New Series*, eds Fretheim T. Gundel J. K. (Philadelphia: John Benjamins Publishing Company), 47–87. 10.1075/pbns.38.04dah

[B25] Demberg V. Keller F. (2008). Data from eye-tracking corpora as evidence for theories of syntactic processing complexity. *Cognition* 109 193–210. 10.1016/j.cognition.2008.07.008 18930455

[B26] Desmet T. Brysbaert M. De Baecke C. (2002). The correspondence between sentence production and corpus frequencies in modifier attachment. *Q. J. Exp. Psychol. Sect. A* 55 879–896. 10.1080/02724980143000604 12188518

[B27] Desmet T. De Baecke C. Drieghe D. Brysbaert M. Vonk W. (2006). Relative clause attachment in Dutch: On-line comprehension corresponds to corpus frequencies when lexical variables are taken into account. *Lang. Cogn. Process.* 21 453–485. 10.1080/01690960400023485

[B28] Dowty D. (1991). Thematic Protoroles-Roles and Argument Selection. *Language* 67 547–619. 10.1353/lan.1991.0021 34409987

[B29] Du Bois J. (2003). *Preferred Argument Structure: Grammar as Architecture for Function.* Amsterdam: John Benjamins. 10.1075/sidag.14

[B30] Farmer T. A. Monaghan P. Misyak J. B. Christiansen M. H. (2011). Phonological typicality influences sentence processing in predictive contexts: Reply to Staub, Grant, Clifton, and Rayner (2009). *J. Exp. Psychol. Learn. Mem. Cogn.* 37 1318–1325. 10.1037/a0023063 21895396

[B31] Feleki E. Branigan H. (1999). “Conceptual accessibility and serial order in Greek speech production,” in *Proceedings of the 21st Annual Conference of the Cognitive Science Society*, eds Hahn M. Stones S. C. (Mahaw, NJ: Lawrence Erlbaum Associates), 96–101. 10.4324/9781410603494-22

[B32] Ferreira F. (2003). The misinterpretation of noncanonical sentences. *Cogn. Psych.* 47 164–203. 10.1016/S0010-0285(03)00005-7 12948517

[B33] Ferreira F. Clifton C. (1986). The independence of syntactic processing. *J. Mem. Lang.* 25 348–368. 10.1016/0749-596X(86)90006-9

[B34] Ferreira V. S. Yoshita H. (2003). Given-New Ordering Effects on the Production of Scrambled Sentences in Japanese. *J. Psycholinguist. Res.* 32 669–692. 10.1023/A:1026146332132 14653013

[B35] Fine A. B. Jaeger T. F. Farmer T. A. Qian T. (2013). Rapid Expectation Adaptation During Syntactic Comprehension. *PLoS One* 8:e77661. 10.1371/journal.pone.0077661 24204909 PMC3813674

[B36] Fine A. B. Qian T. Jaeger T. F. Jacobs R. A. (2010). “Is There Syntactic Adaptation in Language Comprehension?,” in *Proceedings of the 2010 Workshop on Cognitive Modeling and Computational Linguistics CMCL ’10*, (Stroudsburg,PA: Association for Computational Linguistics), 18–26.

[B37] Frank S. L. Bod R. (2011). Insensitivity of the Human Sentence-Processing System to Hierarchical Structure. *Psychol. Sci.* 22 829–834. 10.1177/0956797611409589 21586764

[B38] Frank S. L. Haselager W. (2006). “Robust semantic systematicity and distributed representations in a connectionist model of sentence comprehension,” in *Proceedings of the 28th annual conference of the Cognitive Science Society*, eds Sun R. Miyake N. (Mahwaw, NJ: Lawrence Erlbaum Associates), 226–231.

[B39] Frank S. L. Yang J. (2018). Lexical representation explains cortical entrainment during speech comprehension. *PLoS One* 13:e0197304. 10.1371/journal.pone.0197304 29771964 PMC5957381

[B40] Frank S. L. Otten L. J. Galli G. Vigliocco G. (2015). The ERP response to the amount of information conveyed by words in sentences. *Brain Lang.* 140 1–11. 10.1016/j.bandl.2014.10.006 25461915

[B41] Fraundorf S. H. Jaeger T. F. (2016). Readers generalize adaptation to newly-encountered dialectal structures to other unfamiliar structures. *J. Mem. Lang.* 91 28–58. 10.1016/j.jml.2016.05.006 28377640 PMC5376074

[B42] Frenzel S. Schlesewsky M. Bornkessel-Schlesewsky I. (2011). Conflicts in language processing: A new perspective on the N400–P600 distinction. *Neuropsychologia* 49 574–579. 10.1016/j.neuropsychologia.2010.12.003 21145903

[B43] Friston K. (2010). The free-energy principle: a unified brain theory? *Nat. Rev. Neurosci.* 11 127–138. 10.1038/nrn2787 20068583

[B44] Garnsey S. M. Pearlmutter N. J. Myers E. Lotocky M. A. (1997). The Contributions of Verb Bias and Plausibility to the Comprehension of Temporarily Ambiguous Sentences. *J. Mem. Lang.* 37 58–93. 10.1006/jmla.1997.2512

[B45] Gelman A. (2006). Prior distributions for variance parameters in hierarchical models (comment on article by Browne and Draper). *Bayesian Anal.* 1 515–534. 10.1214/06-BA117A

[B46] Gelman A. Hwang J. Vehtari A. (2014). Understanding predictive information criteria for Bayesian models. *Stat. Comput.* 24 997–1016. 10.1007/s11222-013-9416-2

[B47] Gelman A. Jakulin A. Pittau M. G. Su Y.-S. (2008). A weakly informative default prior distribution for logistic and other regression models. *Ann. Appl. Stat.* 2 1360–1383. 10.1214/08-AOAS191

[B48] Gennari S. P. MacDonald M. C. (2008). Semantic indeterminacy in object relative clauses. *J. Mem. Lang.* 58 161–187. 10.1016/j.jml.2007.07.004 19724662 PMC2735264

[B49] Gibson E. Schütze C. T. Salomon A. (1996). The relationship between the frequency and the processing complexity of linguistic structure. *J. Psycholinguist. Res.* 25 59–92. 10.1007/BF01708420 8789367

[B50] Hale J. (2001). “A Probabilistic Earley Parser as a Psycholinguistic Model,” in *Proceedings of NAACL*, (Pittsburgh, PA: NAACL), 159–166. 10.3115/1073336.1073357

[B51] Harrington Stack C. M. James A. N. Watson D. G. (2018). A failure to replicate rapid syntactic adaptation in comprehension. *Mem. Cognit.* 46 864–877. 10.3758/s13421-018-0808-6 29651687

[B52] Hörberg T. (2016). *Probabilistic and Prominence-driven Incremental Argument Interpretation in Swedish.* Ph. D. thesis. Stockholm: Stockholm University.

[B53] Hörberg T. (2018). Functional motivations behind direct object fronting in written Swedish: A corpus-distributional account. *Glossa* 3:81. 10.5334/gjgl.502

[B54] Hörberg T. Koptjevskaja-Tamm M. Kallioinen P. (2013). The neurophysiological correlate to grammatical function reanalysis in Swedish. *Lang. Cogn. Process.* 28 388–416. 10.1080/01690965.2011.651345

[B55] Hsiao Y. MacDonald M. C. (2016). Production predicts comprehension: Animacy effects in Mandarin relative clause processing. *J. Mem. Lang.* 89 87–109. 10.1016/j.jml.2015.11.006

[B56] Itti L. Baldi P. (2009). Bayesian surprise attracts human attention. *Vision Res.* 49 1295–1306. 10.1016/j.visres.2008.09.007 18834898 PMC2782645

[B57] Jaeger T. F. Norcliffe E. J. (2009). The Cross-linguistic Study of Sentence Production. *Lang. Linguist. Compass* 3 866–887. 10.1111/j.1749-818X.2009.00147.x

[B58] Jegerski J. (2014). “Self-paced reading,” in *Research methods in second language psycholinguistics*, eds Jegerski J. van Patten B. (New York, NY: Routledge), 20–49. 10.4324/9780203123430

[B59] Jurafsky D. (1996). A probabilistic model of lexical and syntactic access and disambiguation. *Cogn. Sci.* 20 137–194. 10.1207/s15516709cog2002_1

[B60] Just M. A. Carpenter P. A. (1980). A Theory of Reading: From Eye Fixations to Comprehension. *Psychol. Rev.* 87 329–354. 10.1037/0033-295X.87.4.329 7413885

[B61] Just M. A. Carpenter P. A. (1992). A Capacity Theory of Comprehension: Individual Differences in Working Memory. *Psychol. Rev.* 99 122–149. 10.1037/0033-295X.99.1.122 1546114

[B62] Kaiser E. Trueswell J. (2004). The role of discourse context in the processing of a flexible word-order language. *Cognition* 94 113–147. 15582623 10.1016/j.cognition.2004.01.002

[B63] Kamide Y. Altmann G. T. M. Haywood S. L. (2003). The time-course of prediction in incremental sentence processing: Evidence from anticipatory eye movements. *J. Mem. Lang.* 49 133–156. 10.1016/S0749-596X(03)00023-8

[B64] Kaschak M. P. Glenberg A. M. (2004). This construction needs learned. *J. Exp. Psychol. Gen.* 133 450–467. 10.1037/0096-3445.133.3.450 15355149

[B65] Kempe V. MacWhinney B. (1999). Processing of Morphological and Semantic Cues in Russian and German. *Lang. Cogn. Process.* 14 129–171. 10.1080/016909699386329

[B66] Kempen G. Harbusch K. (2004). A corpus study into word order variation in German subordinate clauses: Animacy affects linearization independently of grammatical function assignment. *TRENDS Linguist. Stud. Monogr.* 157 173–182. 10.1515/9783110894028.173

[B67] Kennison S. M. (2001). Limitations on the use of verb information during sentence comprehension. *Psychon. Bull. Rev.* 8 132–138. 10.3758/BF03196149 11340858

[B68] Kim A. Osterhout L. (2005). The independence of combinatory semantic processing: Evidence from event-related potentials. *J. Mem. Lang.* 52 205–225. 10.1016/j.jml.2004.10.002

[B69] Kliegl R. Hohenstein S. Yan M. McDonald S. A. (2013). How preview space/time translates into preview cost/benefit for fixation durations during reading. *Q. J. Exp. Psychol.* 66, 581–600. 10.1080/17470218.2012.658073 22515948

[B70] Kretzschmar F. Bornkessel-Schlesewsky I. Staub A. Roehm D. Schlesewsky M. (2012). “Prominence Facilitates Ambiguity Resolution: On the Interaction Between Referentiality, Thematic Roles and Word Order in Syntactic Reanalysis,” in *Case, Word Order and Prominence*, eds Lamers M. de Swart P. (Dordrecht: Springer Netherlands), 239–271. 10.1007/978-94-007-1463-2_11

[B71] Kruschke J. K. (2014). *Doing Bayesian data analysis: a tutorial with R, JAGS, and Stan.* Florida, FL: Academic Press. 10.1016/B978-0-12-405888-0.00008-8

[B72] Kuperberg G. R. (2007). Neural mechanisms of language comprehension: Challenges to syntax. *Brain Res.* 1146 23–49. 10.1016/j.brainres.2006.12.063 17400197

[B73] Kuperberg G. R. Jaeger T. F. (2016). What do we mean by prediction in language comprehension? *Lang. Cogn. Neurosci.* 31 32–59. 10.1080/23273798.2015.1102299 27135040 PMC4850025

[B74] Kuperberg G. R. Caplan D. Sitnikova T. Eddy M. Holcomb P. J. (2006). Neural correlates of processing syntactic, semantic, and thematic relationships in sentences. *Lang. Cogn. Process.* 21 489–530. 10.1080/01690960500094279

[B75] Kuperberg G. R. Kreher D. A. Sitnikova T. Caplan D. N. Holcomb P. J. (2007). The role of animacy and thematic relationships in processing active English sentences: Evidence from event-related potentials. *Brain Lang.* 100 223–237. 10.1016/j.bandl.2005.12.006 16546247

[B76] Kuperberg G. R. Sitnikova T. Caplan D. Holcomb P. J. (2003). Electrophysiological distinctions in processing conceptual relationships within simple sentences. *Cogn. Brain Res.* 17 117–129. 10.1016/S0926-6410(03)00086-7 12763198

[B77] Levy R. (2008). Expectation-based syntactic comprehension. *Cognition* 106 1126–1177. 10.1016/j.cognition.2007.05.006 17662975

[B78] Levy R. (2011). “Integrating surprisal and uncertain-input models in online sentence comprehension: formal techniques and empirical results,” in *Proceedings of the 49th Annual Meeting of the Association for Computational Linguistics: Human Language Technologies-Volume 1*, (Pennsylvania, PA: Association for Computational Linguistics), 1055–1065.

[B79] Lewandowski D. Kurowicka D. Joe H. (2009). Generating random correlation matrices based on vines and extended onion method. *J. Multivar. Anal.* 100 1989–2001. 10.1016/j.jmva.2009.04.008

[B80] Linzen T. Jaeger T. F. (2014). “Investigating the role of entropy in sentence processing,” in *Proceedings of the Fifth Workshop on Cognitive Modeling and Computational Linguistics*, (Pennsylvania, PA: Association for Computational Linguistics), 10–18. 10.3115/v1/W14-2002

[B81] MacDonald M. C. (2013). How language production shapes language form and comprehension. *Front. Psychol.* 4:00226. 10.3389/fpsyg.2013.00226 23637689 PMC3636467

[B82] MacDonald M. C. Seidenberg M. S. (2006). “Constraint Satisfaction Accounts of Lexical and Sentence Comprehension,” in *Handbook of Psycholinguistics*, eds Traxler M. J. Gernsbacher M. A. (Amsterdam: Elsevier), 581–611. 10.1016/B978-012369374-7/50016-X

[B83] MacDonald M. C. Pearlmutter N. J. Seidenberg M. S. (1994). The Lexical Nature of Syntactic Ambiguity Resolution. *Psychol. Rev.* 10 676–703. 10.1037/0033-295X.101.4.676 7984711

[B84] MacWhinney B. Bates E. (1989). *The crosslinguistic study of sentence processing.* New York, NY: Cambridge University Press.

[B85] MacWhinney B. Bates E. Kliegl R. (1984). Cue Validity and Sentence Interpretation in English, German, and Italian. *J. Verbal Learn. Verbal Behav.* 23 127–150. 10.1016/S0022-5371(84)90093-8

[B86] Mak W. M. Vonk W. Schriefers H. (2006). Animacy in processing relative clauses: The hikers that rocks crush. *J. Mem. Lang.* 54 466–490. 10.1016/j.jml.2006.01.001

[B87] Mak W. M. Vonk W. Schriefers H. (2008). Discourse structure and relative clause processing. *Mem. Cognit.* 36 170–181. 10.3758/MC.36.1.170 18323073

[B88] Makowski D. Ben-Shachar M. Lüdecke D. (2019). bayestestR: Describing Effects and their Uncertainty, Existence and Significance within the Bayesian Framework. *J. Open Source Softw.* 4:1541. 10.21105/joss.01541

[B89] McKoon G. Ratcliff R. (2003). Meaning through syntax: Language comprehension and the reduced relative clause construction. *Psychol. Rev.* 110 490–525. 10.1037/0033-295X.110.3.490 12885112 PMC1403829

[B90] McRae K. Spivey-Knowlton M. J. Tanenhaus M. K. (1998). Modeling the Influence of Thematic Fit (and Other Constraints) in On-Line Sentence Comprehension. *J. Mem. Lang.* 1998 283–312. 10.1006/jmla.1997.2543

[B91] Mitchell D. (1987). “Lexical guidance in human parsing: Locus and processing characteristics,” in *Attention and performance 12: The psychology of reading*, ed. Coltheart M. (London: Lawrence Erlbaum Associates, Inc), 601–618.

[B92] Mitchell D. C. (1984). “An Evaluation of Subject-Paced Reading Tasks and Other Methods for Investigating Immediate Processes in Reading,” in *New Methods in Reading Comprehension Research*, eds Kieras D. E. Just M. A. (London: Lawrence Erlbaum Associates, Inc), 69–89. 10.4324/9780429505379-4

[B93] Muralikrishnan R. Schlesewsky M. Bornkessel-Schlesewsky I. (2015). Animacy-based predictions in language comprehension are robust: Contextual cues modulate but do not nullify them. *Brain Res.* 1608 108–137. 10.1016/j.brainres.2014.11.046 25619551

[B94] Nakano H. Saron C. Swaab T. Y. (2010). Speech and span: Working memory capacity impacts the use of animacy but not of world knowledge during spoken sentence comprehension. *J. Cogn. Neurosci.* 22 2886–2898. 10.1162/jocn.2009.21400 19929760

[B95] Narayanan S. Jurafsky D. (1998). “Bayesian Models of Human Sentence Processing,” in *Proceedings of the 20^*th*^ Annual Conference of the Cognitive Science Society*, eds Gernsbacher M. A. Derry S. J. (Mahaw, NJ: Lawrence Erlbaum Associates, Inc), 752–757.

[B96] Nice K. Y. Dietrich R. (2003). Task sensitivity of animacy effects: evidence from German picture descriptions. *Linguistics* 41:027. 10.1515/ling.2003.027

[B97] Nivre J. Megyesi B. (2007). “Bootstrapping a Swedish Treebank Using Cross-Corpus Harmonization and Annotation Projection,” in *Proceedings of the 6th International Workshop on Treebanks and Linguistic Theories*, eds de Smedt K. Hajič J. Kübler S. (Pennsylvania, PA: Association for Computational Linguistics), 97–102.

[B98] Øvrelid L. (2004). “Disambiguation of syntactic functions in Norwegian: modeling variation in word order interpretations conditioned by animacy and definiteness,” in *Proceedings of the 20th Scandinavian Conference of Linguistics*, ed. Karlsson F. (Helsinki: University of Helsinki), 1–17.

[B99] Paczynski M. Kuperberg G. R. (2011). Electrophysiological evidence for use of the animacy hierarchy, but not thematic role assignment, during verb-argument processing. *Lang. Cogn. Process.* 26 1402–1456. 10.1080/01690965.2011.580143 22199415 PMC3244078

[B100] Paczynski M. Kuperberg G. R. (2012). Multiple influences of semantic memory on sentence processing: Distinct effects of semantic relatedness on violations of real-world event/state knowledge and animacy selection restrictions. *J. Mem. Lang.* 67 426–448. 10.1016/j.jml.2012.07.003 23284226 PMC3532895

[B101] Philipp M. Bornkessel-Schlesewsky I. Bisang W. Schlesewsky M. (2008). The role of animacy in the real time comprehension of Mandarin Chinese: Evidence from auditory event-related brain potentials. *Brain Lang.* 105 112–133. 10.1016/j.bandl.2007.09.005 17996287

[B102] Philipp M. Graf T. Kretzschmar F. Primus B. (2017). Beyond Verb Meaning: Experimental Evidence for Incremental Processing of Semantic Roles and Event Structure. *Front. Psychol.* 8:1806. 10.3389/fpsyg.2017.01806 29163250 PMC5670351

[B103] Pickering M. J. Traxler M. J. Crocker M. W. (2000). Ambiguity Resolution in Sentence Processing: Evidence against Frequency-Based Accounts. *J. Mem. Lang.* 43 447–475. 10.1006/jmla.2000.2708

[B104] Primus B. (2006). “Mismatches in semantic-role hierarchies and the dimensions of role semantics,” in *Semantic Role Universals and Argument Linking: Theoretical, Typological and Psycholinguistic Perspectives*, eds Bornkessel I. Schlesewsky M. Comrie B. Friederici A. D. (Berlin: Mouton de Gruyter), 53–89.

[B105] R Core Team (2020). *R: A Language and Environment for Statistical Computing.* Vienna: R Foundation for Statistical Computing.

[B106] Rabovsky M. (2020). Change in a probabilistic representation of meaning can account for N400 effects on articles: A neural network model. *Neuropsychologia* 143:107466. 10.1016/j.neuropsychologia.2020.107466 32315697

[B107] Rabovsky M. Hansen S. S. McClelland J. L. (2018). Modelling the N400 brain potential as change in a probabilistic representation of meaning. *Nat. Hum. Behav.* 2 693–705. 10.1038/s41562-018-0406-4 31346278

[B108] Rahkonen M. (2006). Some aspects of topicalization in Swedish declaratives. *Linguistics* 44 23–55. 10.1515/LING.2006.002

[B109] Ranganath C. Rainer G. (2003). Neural mechanisms for detecting and remembering novel events. *Nat. Rev. Neurosci.* 4 193–202. 10.1038/nrn1052 12612632

[B110] Roark B. Bachrach A. Cardenas C. Pallier C. (2009). “Deriving lexical and syntactic expectation-based measures for psycholinguistic modeling via incremental top-down parsing,” in *Proceedings of the 2009 Conference on Empirical Methods in Natural Language Processing*, (Singapore: Association for Computational Linguistics), 324–333. 10.3115/1699510.1699553

[B111] Roehm D. Bornkessel-Schlesewsky I. Rösler F. Schlesewsky M. (2004). Fractionating language comprehension via frequency characteristics of the human EEG. *J. Cogn. Neurosci.* 15 409–412. 10.1097/00001756-200403010-00005 15094493

[B112] Sauppe S. (2017). Symmetrical and asymmetrical voice systems and processing load: Pupillometric evidence from sentence production in Tagalog and German. *Language* 93 288–313. 10.1353/lan.2017.0015 34409987

[B113] Smith N. J. Levy R. (2013). The effect of word predictability on reading time is logarithmic. *Cognition* 128 302–319. 10.1016/j.cognition.2013.02.013 23747651 PMC3709001

[B114] Spivey-Knowlton M. J. Tanenhaus M. K. (1998). Syntactic ambiguity resolution in discourse: Modeling the effects of referential context and lexical frequency. *J. Exp. Psychol. Learn. Mem. Cogn.* 24 1521–1543. 10.1037/0278-7393.24.6.1521 9835064

[B115] Stan Development Team (2017). *Stan Modeling Language: User’s Guide and Reference Manual.* Columbia: Columbia University.

[B116] Szewczyk J. M. Schriefers H. (2011). Is animacy special? *Brain Res.* 1368 208–221. 10.1016/j.brainres.2010.10.070 21029726

[B117] Szewczyk J. M. Schriefers H. (2013). Prediction in language comprehension beyond specific words: An ERP study on sentence comprehension in Polish. *J. Mem. Lang.* 68 297–314. 10.1016/j.jml.2012.12.002

[B118] Tabor W. Juliano C. Tanenhaus M. K. (1997). Parsing in a dynamical system: An attractor-based account of the interaction of lexical and structural constraints in sentence processing. *Lang. Cogn. Process.* 12 211–271. 10.1080/016909697386853

[B119] Tanaka M. N. Branigan H. P. McLean J. F. Pickering M. J. (2011). Conceptual influences on word order and voice in sentence production: Evidence from Japanese. *J. Mem. Lang.* 65 318–330. 10.1016/j.jml.2011.04.009

[B120] Tily H. (2010). *The Role of Processing Complexity in Word Order Variation and Change.* Ph. D. thesis. Stanford, CA: Stanford University.

[B121] Tooley K. M. Traxler M. J. (2018). Implicit learning of structure occurs in parallel with lexically-mediated syntactic priming effects in sentence comprehension. *J. Mem. Lang.* 98 59–76. 10.1016/j.jml.2017.09.004 29379224 PMC5786270

[B122] Tooley K. M. Swaab T. Y. Boudewyn M. A. Zirnstein M. Traxler M. J. (2014). Evidence for priming across intervening sentences during on-line sentence comprehension. *Lang. Cogn. Neurosci.* 29 289–311. 10.1080/01690965.2013.770892 24678136 PMC3963292

[B123] Traxler M. J. Williams R. S. Blozis S. A. Morris R. K. (2005). Working memory, animacy, and verb class in the processing of relative clauses. *J. Mem. Lang.* 53 204–224. 10.1016/j.jml.2005.02.010

[B124] Trueswell J. C. Tanenhaus M. K. Garnsey S. M. (1994). Semantic Influences on Parsing: Use of Thematic Role Information in Syntactic Ambiguity Resolution. *J. Mem. Lang.* 1994 285–318. 10.1006/jmla.1994.1014

[B125] Van Gompel R. P. G. Pickering M. J. (2001). Lexical guidance in sentence processing: A note on Adams, Clifton, and Mitchell (1998). *Psychon. Bull. Rev.* 8 851–857. 10.3758/BF03196228 11848610

[B126] van Herten M. Chwilla D. J. Kolk H. H. J. (2006). When Heuristics Clash with Parsing Routines: ERP Evidence for Conflict Monitoring in Sentence Perception. *J. Cogn. Neurosci.* 18 1181–1197. 10.1162/jocn.2006.18.7.1181 16839291

[B127] van Herten M. Kolk H. H. J. Chwilla D. J. (2005). An ERP study of P600 effects elicited by semantic anomalies. *Cogn. Brain Res.* 22 241–255. 10.1016/j.cogbrainres.2004.09.002 15653297

[B128] Van Valin R. D. J. (2005). *Exploring the Syntax-Semantics Interface.* Cambridge: Cambridge University Press. 10.1017/CBO9780511610578

[B129] Van Valin R. D. (2006). “Semantic macroroles and language processing,” in *Semantic Role Universals and Argument Linking: Theoretical, Typological, and Psycholinguistic Perspectives*, eds. Bornkessel I. Schlesewsky M. Comrie B. Friederici A. D. (Berlin: Mouton de Gruyter), 263–301.

[B130] Van Valin R. D. J. LaPolla R. J. (1997). *Syntax: Structure, meaning and function.* Cambridge: Cambridge University Press. 10.1017/CBO9781139166799

[B131] Vehtari A. Gelman A. Gabry J. (2017). Practical Bayesian model evaluation using leave-one-out cross-validation and WAIC. *Stat. Comput.* 27 1413–1432. 10.1007/s11222-016-9696-4

[B132] Vosse T. Kempen G. (2000). Syntactic structure assembly in human parsing: a computational model based on competitive inhibition and a lexicalist grammar. *Cognition* 75 105–143. 10.1016/S0010-0277(00)00063-9 10771275

[B133] Vosse T. Kempen G. (2009). The Unification Space implemented as a localist neural net: predictions and error-tolerance in a constraint-based parser. *Cogn. Neurodyn.* 3 331–346. 10.1007/s11571-009-9094-0 19784798 PMC2777195

[B134] Wagenmakers E.-J. (2007). A practical solution to the pervasive problems of *p*-values. *Psychon. Bull. Rev.* 14 779–804. 10.3758/BF03194105 18087943

[B135] Wang L. Wlotko E. Alexander E. Schoot L. Kim M. Warnke L. (2020). Neural Evidence for the Prediction of Animacy Features during Language Comprehension: Evidence from MEG and EEG Representational Similarity Analysis. *J. Neurosci.* 40 3278–3291. 10.1523/JNEUROSCI.1733-19.2020 32161141 PMC7159896

[B136] Warren T. Gibson E. (2002). The influence of referential processing on sentence complexity. *Cognition* 85 79–112. 10.1016/S0010-0277(02)00087-2 12086714

[B137] Watanabe S. (2013). A Widely Applicable Bayesian Information Criterion. *J. Mach. Learn Res.* 14 867–897.

[B138] Weckerly J. Kutas M. (1999). An electrophysiological analysis of animacy effects in the processing of object relative sentences. *Psychophysiology* 36 559–570. 10.1111/1469-8986.3650559 10442024

[B139] Weissbart H. Kandylaki K. D. Reichenbach T. (2020). Cortical Tracking of Surprisal during Continuous Speech Comprehension. *J. Cogn. Neurosci.* 32 155–166. 10.1162/jocn_a_01467 31479349

[B140] Willems R. M. Frank S. L. Nijhof A. D. Hagoort P. van den Bosch A. (2016). Prediction During Natural Language Comprehension. *Cereb. Cortex* 26 2506–2516. 10.1093/cercor/bhv075 25903464

[B141] Wu F. Kaiser E. Andersen E. (2010). “Subject Preference, Head Animacy and Lexical Cues: A Corpus Study of Relative Clauses in Chinese,” in *Processing and Producing Head-final Structures Studies in Theoretical Psycholinguistics*, eds Yamashita H. Hirose Y. Packard J. L. (Dordrecht: Springer Netherlands), 173–193. 10.1007/978-90-481-9213-7_9

[B142] Yan S. Jaeger T. F. (2020). Expectation adaptation during natural reading. *Lang. Cogn. Neurosci.* 35 1394–1422. 10.1080/23273798.2020.1784447

[B143] Yan S. Kuperberg G. R. Jaeger T. F. (2017). Prediction (Or Not) During Language Processing. A Commentary On Nieuwland et al. (2017) And Delong et al. (2005). *bioRxiv* 2017:143750. 10.1101/143750

