# Supplementary material for: A Rational Model of Incremental Argument Interpretation: The Comprehension of Swedish Transitive Clauses
Source: Front Psychol. 2021 Oct 15;12:674202. doi: 10.3389/fpsyg.2021.674202 (PMC8554243; doi:10.3389/fpsyg.2021.674202)
Supplement: Supplementary file 1 [file Data_Sheet_1.PDF]

Supplementary Material

1 Corpus properties

In order to estimate the probability of various argument assignments depending on the linguistic properties of a sentence, we developed an annotated corpus of transitive clauses in written Swedish.

1.1 Sentence materials

The original transitive sentences of the corpus used in this study was collected from the Svensk Trädbank, a syntactically annotated version of the Stockholm-Umeå Corpus (SUC) (Gustafson-Capková & Hartmann, 2006) with materials from the Talbanken (TB) corpus (Einarsson 1976a; b). The SUC corpus consists of 500 published texts from various genres that were published in between 1990 to 1993. The TB corpus consist of 85 professional prose texts from four different genres, written in between 1960 to 1971, originally compiled for the study of Westman (1974). Most of the texts in these two corpora have undergone editing and proofreading. A list of the main genres of the corpora as well as the number of texts and words of each genre is shown in Table S1. The treebank is morphologically and syntactically annotated in Tiger-XML format.

Supplementary Table S1. SUC and TB corpora main genres and their respective text, sentence and word frequencies. Number of search hits in each genre is also shown.

Corpus	Genre	N texts	N sentences	N words	N hits
SUC	Press: reportage	44	7278	106079	1495
	Press: Editorial	17	2385	40887	473
	Press: Reviews	27	3961	66002	712
	Skills, Trades and Hobbies	58	8933	134947	1840
	Popular Lore	48	6525	109665	1503
	Belles Letters, Biography, Memoirs	26	3598	61297	805
	Miscellaneous	70	10847	163333	1540
	Learned and Scientific Writing	83	9633	192827	1809
	General fiction	82	13028	191507	3110
	Mysteries and Science fiction	19	4070	45321	826
	Light reading	20	2908	46126	749
	Humor	6	1071	14428	248
TB	Brochure texts	25	1733	23122	390
	Newspaper texts	28	1669	24125	361
	Educational texts	14	1624	25623	374
	Debate articles	18	1134	23476	316

1.2 Corpus searches

Searches were conducted with TIGER search 2.1. (König et al. 2003) with search strings in the TIGER search query language (König & Lezius 2003). Three types of search patterns were used to find the three general syntactic patterns of subject-initial (SVO), object-initial (OVS) and adverbial initial sentences (VSO/VOS). These search patterns are exemplified in Table S2. The actual search strings can be provided upon request. The patterns contain noun phrases (NP), adverb phrases (AdvP), verbs (V1, V2, V3, V4), verb particles (PT), and topicalized adverbial phrases (TP) which could consist of nominal, adverb or prepositional phrases. The initial verbs (V1) were always finite,

Supplementary Table S2. The three general syntactic patterns corresponding to the search patterns searching for SVO, OVS and VSO sentences. Optional constituents / words are shown in parentheses. NP = Nominal Phrase; AdvP = Adverbial Phrase; V = Verb (V_1 = finite; V_2 = infinite or infinitival marker *att*; V_3 + V_4 = Infinite); PT = Verb particle.

	**Search pattern**
**SVO**	[NP] ([ADVP]) ([ADVP]) [V$_1$] ([ADVP]) ([ADVP]) ([V$_2$]) ([V$_3$]) ([V$_4$]) ([PT]) [NP]
	[Du som har examen enligt den tidigare studieordningen och uppfyller de nya kraven för en magisterexamen$_{NP}$] [kommer$_{V1}$] [att$_{V2}$] [kunna$_{V3}$] [ta$_{V4}$] [ut$_{PT}$] [en sådan$_{NP}$].
	You who have a degree according to the previous curriculum and is eligible for a magister's degree will be able to acquire such.
**OVS**	[NP] [V$_1$] ([ADVP]) ([ADVP]) [NP] ([ADVP]) ([ADVP]) [V$_2$] ([V$_3$]) ([V$_4$]) ([PT])
	[Några egentliga genombrott från de informella överläggningarna ute på Bushs lantställe Camp David$_{NP}$] [kunde$_{V1}$] [de$_{NP}$] [inte$_{ADVP}$] [redovisa$_{V2}$].
	Any serious breakthroughs from de informal negotiations at Bush's country house Camp David they were unable to report.
**VSO/ VOS**	[TP] [V1] ([ADVP]) ([ADVP]) [NP] ([PT]) ([ADVP]) ([ADVP]) ([V2]) ([V3]) ([V4]) ([PT]) [NP]
	[Möjligen$_{TP}$] [kommer$_{V1}$] [han$_{NP}$] [till och med$_{ADVP}$] [att$_{V2}$] [älska$_{V3}$] [denna hans, Lauritz', stora handling$_{NP}$].
	Possibly will he even love this his Lauritz´ big action.

the second verb (V2) was either an infinite verb or an infinitival marker, and the final verbs (V3 and V4) were infinite. In the table, optional elements are shown in parentheses.

Due to inconsistencies and errors in the syntactic annotation of the corpus, search strings relied on both hierarchical dependencies and linear order. Multi-word constituents were defined with reference to the directly dominating node as well as to the left- and right-most word nodes dominated by the constituent node at hand. Consecutive search queries that defined syntactic relationships between constituents would then make reference to precedence relations between either the right- or the left-most word node of the constituent node at hand and some other node. In order to allow for the structural variation shown in Table S2, the search queries contained disjunctions of alternative search patterns that were organized in a nested fashion. For example, search patterns for subject-initial sentences without adverbial phrases had the following simplified structure:

[NP1] [V1] & (([V1] [NP2] | [V1] [PL] [NP2])

| [V1] [V2] & (([V2] [NP2] | [V2] [PL] [NP2])

| [V2] [V3] & ([V3] [NP2] | [V3] [PL] [NP2])

| [V3] [V4] & ([V4] [NP2] | [V4] [PL] [NP2]))))

Which is equivalent to the following pattern, in which () represent optional elements:

[NP1] [V1] ([V2]) ([V3]) ([V4]) ([PL]) [NP2]

The overall intent of the data collection procedure was to find as many of the transitive sentences available in the corpus as possible, with the aim to minimize the constraints on the structural variation of the sentences. Search patterns were therefore constructed so as to avoid false exclusions

rather than false inclusions. False hits were then manually excluded from the initial data. Excluded clause types are listed in Supplementary Table S3.

Supplementary Table S3. Excluded clause types and example sentences of each type.

**Clause type**		**Example**
direct question		Var har [magin$_{NP}$] tagit [vägen$_{NP}$] , Morrissey?<br>*Where has the magic gone, Morrissey?*
intransitive sentence		[Sommartid$_{NP}$] går [en buss$_{NP}$] härifrån till Bourg St Maurice.<br>*In the summer a buss departs from here to Bourg St Maurice.*
incorrectly annotated sentence		Eller [rättare sagt$_{*NP}$] återupplivar [en gammal$_{NP}$].<br>*Or rather revive an old one*
non-NP object	clause	[En sådan människa$_{NPi}$] trodde [jag$_{NP}$] [att jag var [__$_{NPi}$]$_{S}$].<br>*Such a person I thought I was.*
	infinitival phrase	När [Aktuellt$_{NP}$] väljer [att följa den linjen$_{InfP}$].<br>*When Aktuellt choose to take that approach.*
dummy argument	Subject	[det$_{NP}$] står [20 studenter$_{NP}$] i korridorerna.<br>*20 students stand in the hallways.*
	object	Tillsammans ska [vi$_{NP}$] bona om [det$_{NP}$] där hemma.<br>*Together we will make it more habitable at home.*
embedded adverbial phrase		… när [han$_{NP}$] [den där höstdagen 1977$_{NP}$] skjutsade [henne$_{NP}$].<br>*... when he in the autumn of 1977 drove her.*
ditransitive sentence	NP	Och [bättre betyg$_{NP}$] kan [jag$_{NP}$] inte ge [mig själv$_{NP}$].<br>*And I can´t give myself a better score.*
	clause	[Jag$_{NP}$] frågar [Jane Landeryou$_{NP}$] [varför hon är emot tjurfäktningar$_{S}$].<br>*I ask Jane Landeryou why she is against bullfights.*
predicative sentence	subject predicative	[Var tredje människa$_{NP}$] är [analfabet$_{NP}$]..<br>*Every third person is illiterate.*
	object predicative	[Hummern$_{NP}$] kallar [Per Moksnes$_{NP}$] för Rocky.<br>*The lobster Per Moksnes calls Rocky.*
	object with infinitive	[Jag$_{NP}$] såg [världen$_{NP}$] gå under.<br>*I saw the world perish.*
	causative sentence	[En hastig ingivelse$_{NP}$] får [mig$_{NP}$] att härma tjuren.<br>*A sudden impulse makes me imitate the bull.*
idiomatic expression	idiom	Även [fack och forskare$_{NP}$] får [sin släng av sleven$_{NP}$].<br>*Also the unions and scientists get theirs.*<br>(lit: Also the unions and scientists get their throw of the trowel)
	lexicalisation	[alla invånare$_{NP}$] [skulle ha [råd$_{NP}$] med [bil$_{NP}$]$_{VP}$].<br>*All citizens would afford a car.*
light constituent	light verb	[Demodokos$_{NP}$] gör [ett besök$_{NP}$] i underjorden.<br>*Demodokos makes a visit to the underworld.*
	light object	[det$_{NP}$] kostar [pengar$_{NP}$].<br>*It costs money.*<br>[Han$_{NP}$] målar [massor$_{NP}$].<br>*He paints a lot.*

1.3 Sentence annotation

All remaining transitive clauses were annotated for the following properties that previous work on Swedish or other languages has identified to affect constituent order: givenness (new vs. given), animacy (inanimate vs. animate), definiteness (indefinite vs. definite), person / egophoricity (1st & 2nd person vs. 3rd person), number (singular vs. plural), pronominality (pronoun vs. noun head), case

(subject form vs. object form vs. unmarked), text deixis[1] (text deictic vs. non-text deictic), constituent length in words (continuous), verb semantics and three syntactic variables. Verb semantic classification was based on the classification of Dowty (1991) and Primus (2006), as illustrated in Table S4. Verb semantic categories were not mutually exclusive. The three syntactic variables included whether the clause was embedded in another clause, whether the clause contained an auxiliary verb, and whether the verb of the clause preceded the NP arguments.

All of these linguistic properties are considered by the models of constituent order that are presented in the next section. The annotation procedure is described in more detail in Hörberg (2016). We note that our corpus also contained sentences in which the verb preceded both of its arguments. Since Swedish is a verb-second language, this can only happen in adverbial-initial clauses. In such clauses, an object-before-subject (OS) order is *highly* infrequent (since it is only possible when the object consists of a weak and unstressed personal pronoun, Hörberg 2016: 38-40, 87; Heinat 2010). There are only three such occurrences in our corpus. We thus annotated whether the verb preceded both of its arguments (Verb before S and O, in Tables S6-8 below), and included this feature in the analyses of constituent order that we present next.

Supplementary Table S4. Verb semantic categories of the main verbs in the sentence materials. Column 1 lists the categories used in the present work whereas column 2 and 3 show how these categories correspond to those of Dowty (1991) and Primus (2006).

**Category**	**Dowty (1991)**		**Primus (2006)**
	**Actor**	**Undergoer**	
**Volitionality**	Volitional involvement	Undergoes change of state	Control – volitionality and intentionality
**Experiencer**	Sentience	-	Sentience
**Causation**	Cause event or change of state<br>Movement	Incremental theme<br>Causally affected<br>Stationary	Physical involvement
**Possession**	-	-	Possession

2 Estimating the probability of OS vs. SO order based on the corpus data

Bayesian mixed-effects logistic regression models (GLMMs) were fitted to the corpus in order to estimate the probability of OS vs. SO order, p(OS), as a function of the linguistic cues available in the sentence. Specifically, four separate models were fit, each capturing the information available at different regions of the sentence. These four models informed the design of our self-paced reading experiment, and were used to predict Bayesian surprise for the stimuli of that experiment:

(1) The **clause onset model** contained only an intercept, thus capturing the overall preference for SO vs. OS at the sentence onset.

(2) The **NP1 model** contained only (and all) cues available in the first NP of the sentence.

[1] That is, whether the NP is a neuter pronominal or demonstrative (i.e., *det* and *detta* – 'that') that refers back to a proposition in the immediate left context. Such NPs frequently occupy the sentence initial position Swedish (Hörberg 2016; 2018). Text deixis thus serves as a highly reliable cue to argument interpretation.

(3) The **NP1 + verb model** contained only (and all) cues available on the first NP and the verb.

(4) Finally, the **NP1 + verb + NP2 model** contained only (and all) cues available in either of the NPs and the verb.

2.1 Fitting the mixed-effects logistic regressions

All GLMMs were fit with the statistical package *brms* (Bürkner, 2017, 2018) in *R* (R Core Team, 2020). The R code is available at https://osf.io/rw5nf/. All models contained random intercepts for genre. All categorical predictors were treatment-coded, though we note that the coding choice does not change the predictions of the GLMM (the linear predictor—the sum of all terms in the model's formula—is invariant under different coding). Verb semantic category was coded by multiple separate semantic features since a verb could be, for example, both volitional and experiencer. We also considered all interactions of NP properties and verb semantics, and used backward elimination to determine which of these interactions had significant effects on constituent order (for details, see Hörberg, 2016: 99).

The GLMMs we fit employed regularizing priors, following recommendations in the regression literature and machine learning. Regularizing priors 'shrink' coefficient estimates towards zero reducing the chance of overfitting to the data, and facilitate model convergence. This leaves open what the *strength* of the prior should be. For a regularizing prior, stronger priors shrink estimates more strongly towards zero, compared to weaker priors. Here, we use GLMM as a (highly simplified) model of the subjective beliefs comprehenders hold about the relative probability of OS vs. SO order. Within the broad type of experienced-based account of language processing, these subjective beliefs (or expectations) are assumed to be the outcome of language acquisition (for review, see MacDonald, 2013; Dell & Chang, 2013; Kuperberg & Jaeger, 2016). More specifically, within the rational framework we operate in here, the subjective beliefs are assumed to have resulted from the rational integration of initial prior beliefs and the statistics observed in the language input. To the extent that the sample of 16,552 sentences we fit the model to here is representative of the statistics in the relevant language input experienced by a typical learner of Swedish, the strength of the regularizing prior can be seen as scaling these 16,552 sentences to the amount of this type of data a typical learner is assumed to have experienced during acquisition. However, since the strength of learners' priors at the onset of acquisition is not known this reasoning cannot be used to constrain the range of plausible priors for the GLMM. We therefore decided to follow recommendations in the literature on data analysis for the random effect priors, and to use weaker regularizing priors for fixed effects (e.g., Gelman, 2006; Gelman et al. 2008; Stan Development Team 2017). Specifically, the analyses presented in the main text use a Cauchy prior with location 0 and scale 2 for the standard deviation of random effects (i.e., the by-genre intercepts). For the intercept, we used a normal prior with mean -2.994 (the log-odds of the overall proportion of OS order, which is .05), and a scale of 2.5. For all other fixed effects, we used Student *t* prior centered at 0 with 30 degrees of freedom and a scale of 5.

2.2 Results: Fitted mixed-effects logistic regressions predicting OS vs. SO order

Tables S5-S8 summarize the four GLMMs after backwards elimination of non-significant interactions between NP properties and verb semantics.

Supplementary Table S5. Fixed effects statistics of the clause onset model.

Parameter	$\hat{\beta}_{MAP}$	S.E.($\hat{\beta}$)	HDPI$_{lower}$	HDPI$_{upper}$	$p_{posterior}$	$\hat{R}$	ESS
Intercept	-3.01	0.08	-3.14	-2.87	1.000	1.00	21806.58

Supplementary Table S6. Fixed effects statistics of the NP1 model.

Parameter	$\hat{\beta}_{MAP}$	S.E.($\hat{\beta}$)	HDPI$_{lower}$	HDPI$_{upper}$	$p_{posterior}$	$\hat{R}$	ESS
Intercept	-4.15	2.33	-7.62	-0.22	0.95	1.00	39323.50
NP1 Animacy	2.68	0.15	2.46	2.94	1.000	1.00	88216.95
NP1 Givenness	-0.23	0.16	-0.50	0.02	0.926	1.00	43283.64
NP1 Definiteness	0.80	0.14	0.57	1.02	1.000	1.00	53408.04
NP1 Number	0.06	0.11	-0.13	0.23	0.675	1.00	90681.52
NP1 Person / Egophoricity	-0.47	2.32	-4.37	2.98	0.629	1.00	39961.79
NP1 Pronominal	0.01	0.17	-0.26	0.29	0.522	1.00	49104.54
NP1 Case: SO	-5.89	2.05	-9.57	-3.41	1.000	1.00	27320.27
NP1 Case: OS	11.81	2.76	8.73	17.12	1.000	1.00	34152.43
NP1 Text Deixis	2.19	0.19	1.91	2.51	1.000	1.00	48566.32
NP1 Length	0.28	0.10	0.13	0.45	0.998	1.00	55838.13
Embedded	-1.56	0.13	-1.78	-1.36	1.000	1.00	89536.56
Verb before S and O	-4.89	1.37	-7.49	-3.44	1.000	1.00	38141.89

Supplementary Table S7. Fixed effects statistics of the NP1 + verb model.

Parameter	$\hat{\beta}_{MAP}$	S.E.($\hat{\beta}$)	HDPI$_{lower}$	HDPI$_{upper}$	$p_{posterior}$	$\hat{R}$	ESS
Intercept	-4.75	2.34	-8.52	-1.13	0.977	1.00	44962.41
NP1 Animacy	2.95	0.24	2.60	3.37	1.000	1.00	62691.47
NP1 Givenness	-0.21	0.22	-0.57	0.12	0.847	1.00	50386.65
NP1 Definiteness	0.70	0.20	0.39	1.02	1.000	1.00	53868.91
NP1 Number	0.03	0.15	-0.21	0.27	0.573	1.00	98231.45
NP1 Person / Egophoricity	-1.32	2.32	-4.78	2.56	0.699	1.00	45144.27
NP1 Pronominal	0.37	0.25	0.00	0.81	0.953	1.00	51577.49
NP1 Case: SO	-4.52	1.92	-8.27	-2.52	1.000	1.00	36285.50
NP1 Case: OS	12.74	2.72	9.35	17.60	1.000	1.00	35707.23
NP1 Text Deixis	2.42	0.27	1.99	2.87	1.000	1.00	50811.70
NP1 Length	0.36	0.13	0.16	0.57	0.997	1.00	68045.45
Volitional	-0.02	0.49	-0.84	0.72	0.537	1.00	43937.03
Experiencer	0.45	2.67	-4.13	4.34	0.542	1.00	37842.52
Causative	0.30	0.77	-1.04	1.33	0.601	1.00	35352.48
Possessive	-0.44	0.22	-0.82	-0.12	0.985	1.00	59038.20
Auxiliary: SO	-5.79	1.68	-8.87	-4.01	1.000	1.00	36302.71
Auxiliary: OS	9.60	2.42	7.45	14.30	1.000	1.00	30736.95
Embedded	-1.50	0.17	-1.77	-1.23	1.000	1.00	97050.90
Verb before S and O	-4.18	1.34	-6.65	-2.69	1.000	1.00	41124.72
NP1 Animacy × Volitional	1.47	0.53	0.63	2.30	0.999	1.00	43724.49
NP1 Person × Experiencer	2.24	2.67	-1.82	6.65	0.832	1.00	37900.74
NP1 Animacy × Causation	0.98	0.79	-0.08	2.38	0.946	1.00	35306.71
NP1 Definiteness × Possessive	1.38	0.33	0.83	1.89	1.000	1.00	56215.71

Supplementary Table S8. Fixed effects statistics of the NP1 + verb + NP2 model.

**Parameter**	$\hat{\beta}_{MAP}$	**S.E.**($\hat{\beta}$)	**HDPI**$_{lower}$	**HDPI**$_{upper}$	$p_{posterior}$	$\hat{R}$	**ESS**
Intercept	-1.35	3.64	-7.15	4.39	0.663	1.00	32592.74
NP1 Animacy	3.58	0.53	2.86	4.53	1.000	1.00	33133.34
NP1 Givenness	-0.72	0.39	-1.35	-0.09	0.966	1.00	35525.35
NP1 Definiteness	1.36	0.36	0.79	1.93	1.000	1.00	39299.04
NP1 Number	0.44	0.27	0.01	0.86	0.953	1.00	63669.36
NP1 Person / Egophoricity	-0.71	2.38	-4.58	2.98	0.644	1.00	32918.34
NP1 Pronominality	0.07	0.52	-0.70	0.95	0.605	1.00	31828.13
NP1 Text Deixis	1.74	0.55	0.93	2.67	1.000	1.00	31400.94
NP1 Case: SO	-3.10	2.00	-6.81	-0.77	0.995	1.00	27033.26
NP1 Case: OS	13.83	2.89	10.23	19.13	1.000	1.00	31164.09
NP1 Length	0.50	0.24	0.11	0.86	0.979	1.00	48522.56
NP2 Animacy	-2.55	0.25	-2.95	-2.17	1.000	1.00	45925.86
NP2 Givenness	-0.35	0.34	-0.92	0.18	0.857	1.00	33005.35
NP2 Definiteness	-1.76	0.32	-2.30	-1.28	1.000	1.00	47673.63
NP2 Number	-0.37	0.24	-0.73	0.04	0.929	1.00	61830.77
NP2 Person / Egophoricity	-1.20	2.65	-5.43	3.00	0.678	1.00	33626.67
NP2 Pronominality	-1.18	0.39	-1.81	-0.55	0.998	1.00	40185.91
NP2 Text Deixis	-0.37	0.82	-1.61	1,00	0.645	1.00	47479.62
NP2 Case: OS	9.06	3.16	5.16	14.59	1.000	1.00	29715.13
NP2 Case: SO	-8.34	2.41	-12.91	-5.74	1.000	1.00	28274.55
NP2 Length	0.08	0.28	-0.38	0.53	0.608	1.00	35011.88
Volitionality	0.54	0.88	-0.96	1.85	0.716	1.00	28042.31
Experiencer	0.42	3.53	-5.39	5.83	0.524	1.00	34203.97
Causation	0.89	1.17	-1.20	2.46	0.721	1.00	26208.46
Possessive	-0.02	1.23	-2.04	1.87	0.527	1.00	29186.67
Auxiliary: SO	-4.91	1.65	-8.01	-3.13	1.000	1.00	30780.57
Auxiliary: OS	9.50	2.58	6.94	14.4	1.000	1.00	31247.35
Embedded	-1.42	0.32	-1.95	-0.93	1.000	1.00	61220.18
Verb before S and O	-2.89	1.42	-5.61	-1.32	1.000	1.00	33435.32
NP1 Animacy × Volitional	1.53	0.98	0.19	3.31	0.970	1.00	27428.75
NP2 Animacy × Volitional	-1.90	0.72	-3.08	-0.80	0.998	1.00	51122.94
NP1 Person × Experiencer	1.70	2.99	-2.40	7.06	0.764	1.00	36363.19
NP2 Person × Experiencer	-0.27	3.09	-5.50	4.34	0.580	1.00	38557.45
NP1 Animacy × Causation	0.23	1.22	-1.27	2.57	0.674	1.00	26658.28
NP1 Definiteness × Possessive	0.52	0.77	-0.65	1.80	0.768	1.00	51587.69
NP2 Givenness × Possessive	-3.30	0.78	-4.59	-2.13	1.000	1.00	55018.61
NP2 Pronominality × Possessive	1.96	1.25	0.05	4.00	0.954	1.00	30012.95

2.3 Assessing effects of the priors on the fit

Post-hoc, to assess whether the robustness of the results reported in the main text, we entertained priors with a range of regularizing strengths (see R code at https://osf.io/rw5nf/). We illustrate the consequences of these different prior strengths for a) the predictive accuracy of the model of OS vs. SO order and b) the resulting estimates of Bayesian surprise.

Regularizing priors pull coefficient estimates towards zero, a result also referred to as “shrinkage”. This shrinkage can reduce the risk of over-fitting the GLMM to the data, and also aids convergence during the fitting of the GLMM. Shrinkage is larger, the less data the model has access to and the stronger the prior. In the context of the rational model, priors with different regularizing strengths can also been seen as representing different hypotheses about how much data a typical adult native speaker of Swedish—i.e., the type of language user that we aim to model—has seen prior to our self-paced reading experiment. Under the assumptions that a) the 16,552 transitive sentences in our corpus are representative of written Swedish and b) the human brain uses similar regularizing priors as the ones we use in the fitting of the GLMMs, stronger regularizing priors can be seen as simulating a scenario in which the typical adult native speaker has seen less previous input, compared to weaker priors (since stronger priors pull the coefficient estimates closer to zero, down-weighting the corpus data).

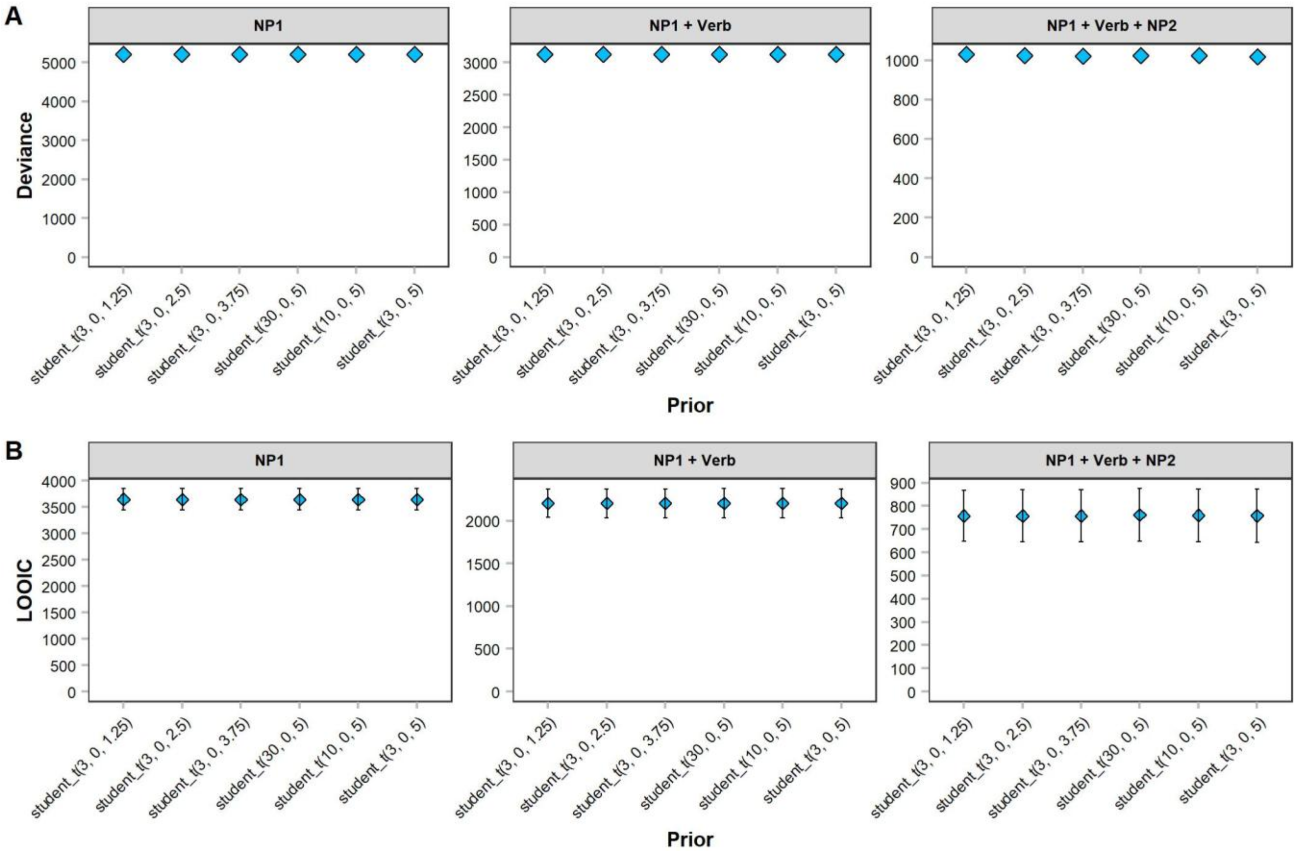

Supplementary Figure S1. Deviance (panel A) and out-of-sample predictive accuracy in terms of LOOIC (panel B) of each of the Bayesian GLMMs used in the rational model of incremental argument interpretation as a function of fixed effects prior strength. We considered Student *t* priors centered at zero with 3 DFs and scales 1.25, 2.5, and 3.375, or with a scale of 5 and DFs of 30, 10 and 3. The regularizing strength of the prior goes down with a *larger* scale, or with a *smaller* number of DFs.

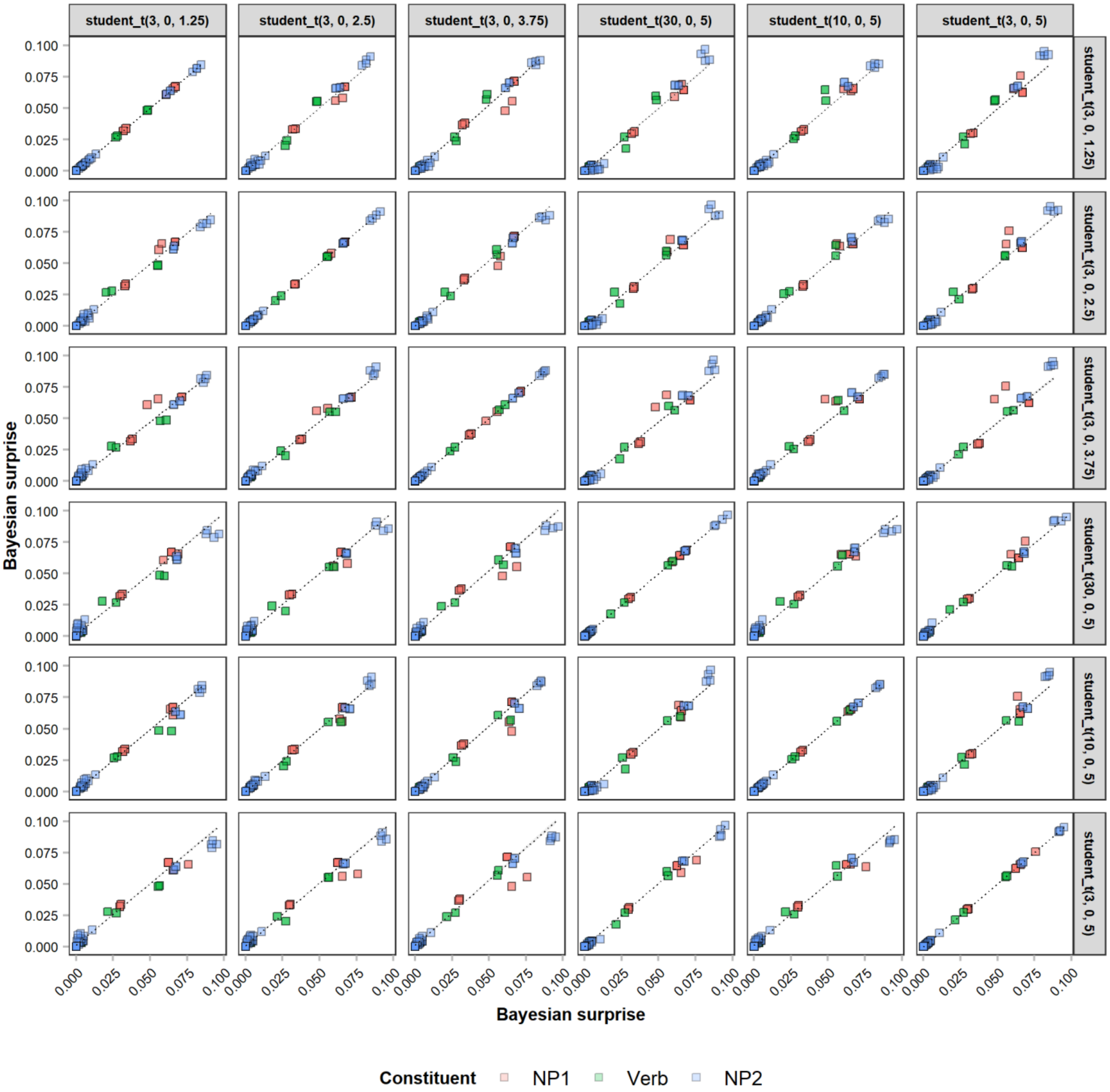

Supplementary Figure S2. Comparisons of Bayesian surprise in subject-initial sentences as predicted by the rational model of incremental argument interpretation model as a function of fixed effects prior strength. We considered Student *t* priors centered at zero with 3 DFs and scales 1.25, 2.5, and 3.375, or with a scale of 5 and DFs of 30, 10 and 3. The regularizing strength of the prior goes down with a *larger* scale, or with a *smaller* number of DFs.

We thus refitted all GLMMs under a range of fixed-effects priors, going from strongly to very weakly regularizing. Specifically, we considered three Student *t* priors centered at zero with 3 degrees of freedom (DFs) and scales of 1.25, 2.5 and 3.375, and three Student *t* priors centered at zero with a scale of 30, but with 30, 10 and 3 DFs. The regularizing strength of these priors go down with a *larger* scale, or with a *smaller* number of DFs. Figure S1 compares GLMMs with respect to their deviance (= -2 * log likelihood) and their out-of-sample predictive ability—their estimated log posterior distribution (elpd), calculated on the basis leave-one-out cross validation (see Vehtari et al. 2013). Supplementary Figures S2 and S3 compare the GLMMs with respect to how they affect predictions of Bayesian surprise, either in subject-initial (Figure S2) or object-initial (Figure S3)

sentences. For the comparisons, all models were fit using 12 chains with 1000 warmup-samples and 4000 post-warmup samples per chain, resulting in 48,000 posterior samples for each analysis.

Choice of priors does not have a big impact, neither in terms of out-of-sample predictions of the underlying GLMMs (Figure S1), nor with respect to Bayesian surprise as predicted by the rational model of argument interpretation (Figures S2 and S3). Given the high correlations between Bayesian surprise estimates derived under a wider range of prior strengths, we did not further explore which of these priors provides the best fit against human reading times. This could be explored in future work.

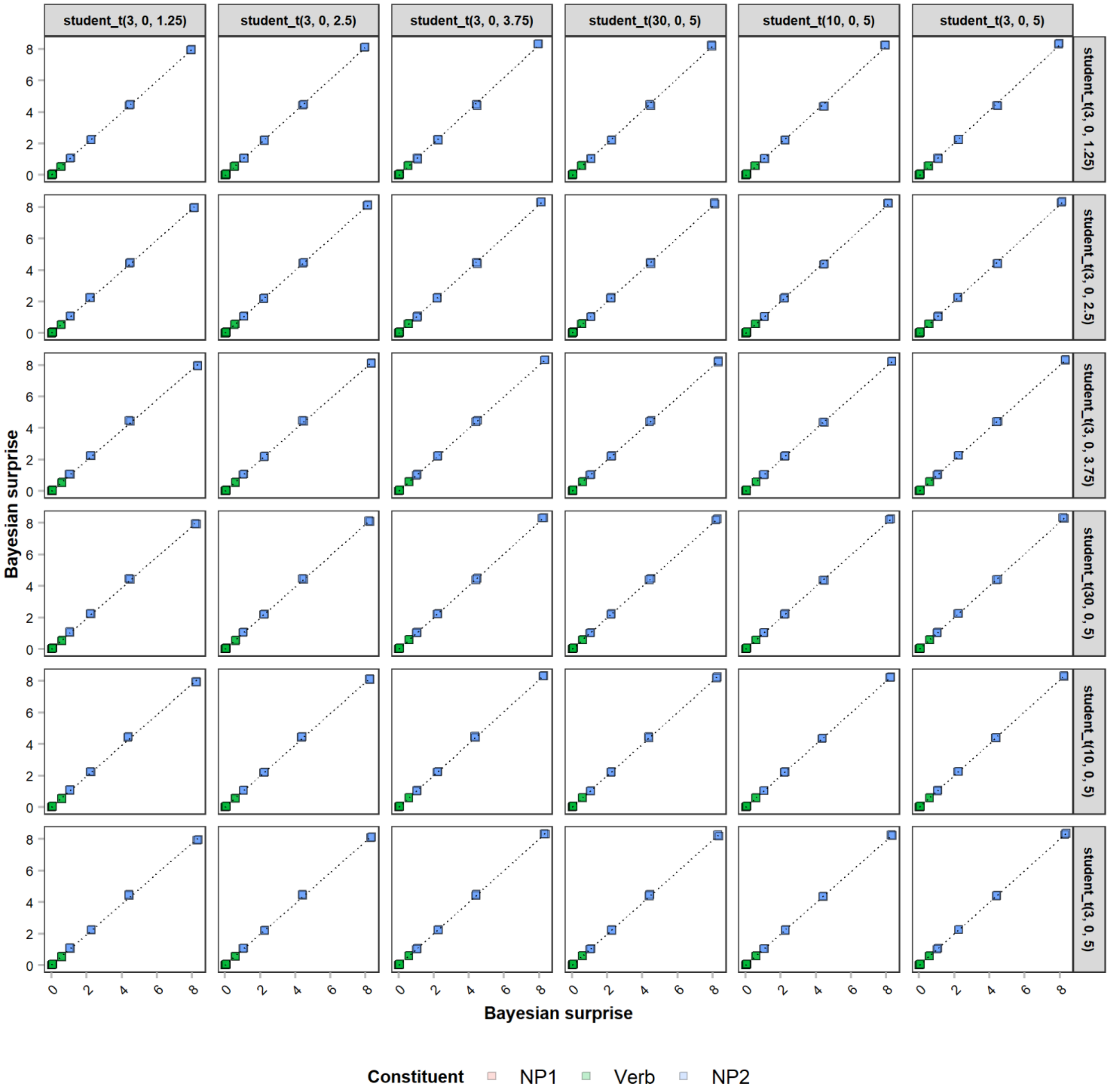

Supplementary Figure S3. Comparisons of Bayesian surprise in object-initial sentences as predicted by the rational model of incremental argument interpretation model as a function of fixed effects prior strength. We considered Student *t* priors centered at zero with 3 DFs and scales 1.25, 2.5, and 3.375, or with a scale of 5 and DFs of 30, 10, 3. The regularizing strength of the prior goes down with a *larger* scale, or with a *smaller* number of DFs.

3 Illustrating the effect of linguistic properties on Bayesian surprise across sentence regions

Additional predictions of the rational model of incremental argument interpretation are illustrated in Figure S4 and Figure S5. Figure S4 illustrates predicted changes in Bayesian surprise in sentences with a lexical NP1 and a 1st person pronoun NP2 as a function of NP1 prominence cues and verb semantic cues, comparing causative and possessive verbs. Figure S4 thus parallels Figure 3 in the main text, but instead compares causative and possessive verbs. The effect of verb class is weaker for these verbs.

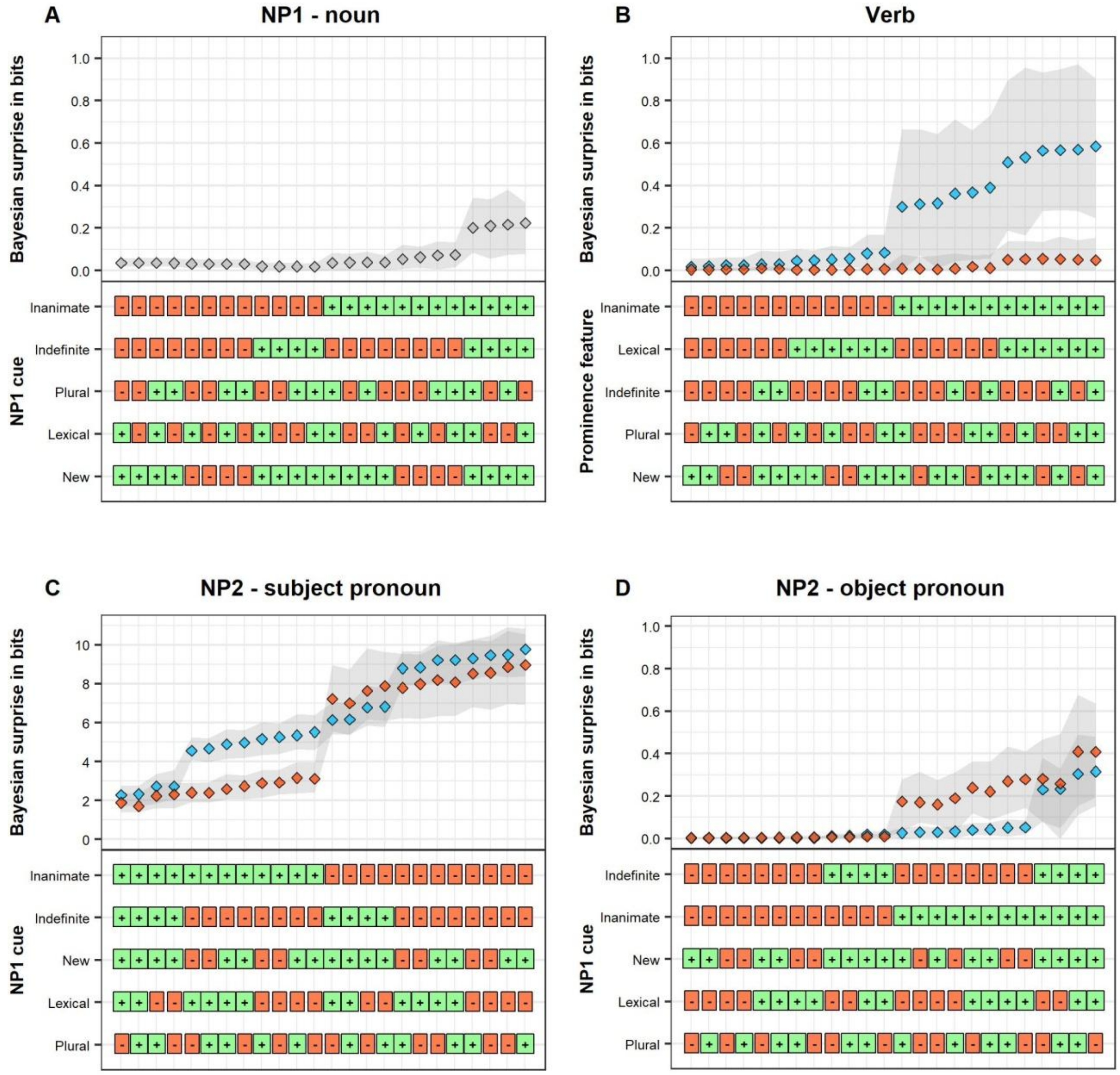

Supplementary Figure S4. Predicted Bayesian surprise of the NP1, verb, and NP2 constituents of a transitive sentence with a lexical NP1 and a 1st person pronoun NP2. Bayesian surprise is shown as a function of NP1 cues (green square with plus indicates presence of feature) and verb semantics (color: red for causative and blue for possessive verbs). Panel A: Bayesian surprise for NP1 (verb semantic information not yet available). Panel B: Bayesian surprise for the verb (causative vs. possessive). Panel C: Bayesian surprise for NP2 when NP2 is a case-marked subject pronoun disambiguating towards OVS order. Panel D: Bayesian surprise for NP2 when NP2 is a case-marked object pronoun, disambiguating towards SVO order. Shaded areas illustrate 89% highest posterior density intervals (HPDIs) of predicted Bayesian surprise, calculated on the basis of the posterior predictions of the underlying Bayesian GLMMs. Note that the range of the y-axis as well as the order of cues differ between plots. For each panel cues are ordered in decreasing importance from top to bottom.

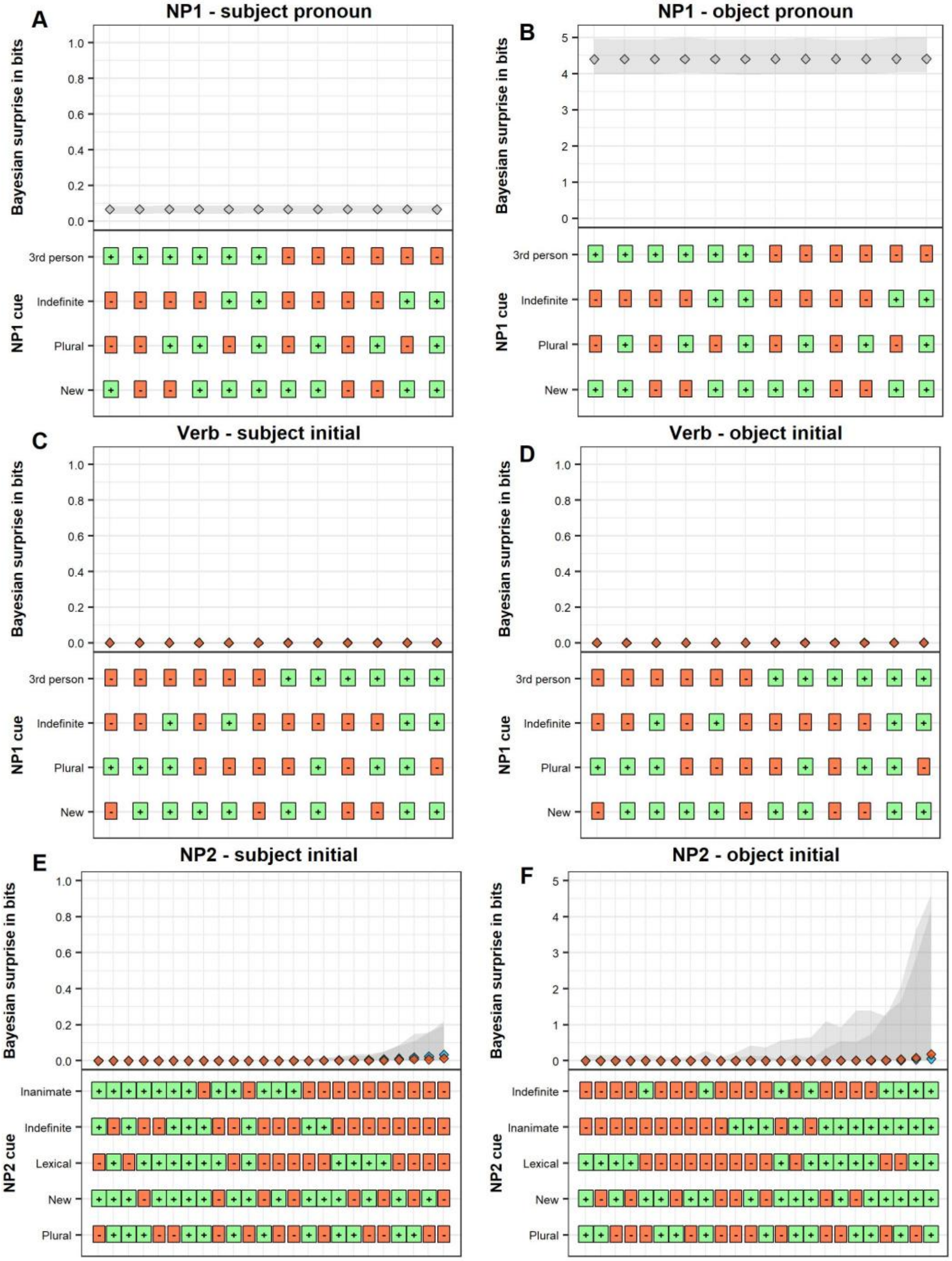

Supplementary Figure S5. Predicted Bayesian surprise in transitive sentences with a 1st person pronoun NP1 and a lexical NP2. Bayesian surprise is shown as a function of NP1 or NP2 cues (green square with plus indicates presence of feature). Panel A: Bayesian surprise at subject-case marked NP1 as a function of NP1 cues. Panel B: Bayesian surprise at object-case marked NP1 as a function of NP1 cues. Panel C: Bayesian surprise at verb in subject-initial sentences as a function of NP1 cues and verb semantics (color: red for volitional and blue for experiencer verbs). Panel D: Bayesian surprise at verb in object-initial sentences as a function of NP1 cues and verb semantics (color: red for volitional and blue for experiencer verbs). Panel E: Bayesian surprise at NP2 in subject-initial sentences as a function of NP2 cues and verb semantics (color: red for volitional and blue for experiencer verbs). Panel F: Bayesian surprise at NP2 in object-initial sentences as a function of NP2 cues and verb semantics (color: red for volitional and blue for experiencer verbs). Shaded areas illustrate 89% highest posterior density intervals (HPDIs) of predicted Bayesian surprise, calculated on the basis of the posterior predictions of the underlying Bayesian GLMMs. Note that the range of the y-axis as well as the order of cues differ between plots. For each panel cues are ordered in decreasing importance from top to bottom.

Figure S5 shows changes in Bayesian surprise with a 1st person pronoun NP1, either with subject (panels A, C and E) or object (panels B, D and F) case-marking, as a function of NP1 (panels A-D) or NP2 (panels E-F) prominence cues. As such, Figure S5 shows Bayesian surprise in unambiguous transitive sentences, in which the sentence at hand is disambiguated either towards SVO (panels A, C and E) or OVS (panel B, C and F) directly at NP1. Unambiguous information regarding argument interpretation is thus provided already at NP1. In subject-initial sentences, this is in line with the baseline assumption of SVO word order, and there is no effect in Bayesian surprise. In object-initial sentences, on the other hand, the sentence-initial object pronoun violates the baseline assumption of SVO word order and there is a large effect of Bayesian surprise. At subsequent constituents, there is no change in the expectation for OVS word order as unambiguous information already has been provided.

4 Lexical items in the self-paced reading experiment

Table S9 lists all the lexical items that were used in each stimulus item in the self-paced reading experiment.

Supplementary Table S9. The nouns, verbs and pronouns used in the self-paced reading experiment.

**Inanimate noun**	**Animate noun**	**Volitional verb**	**Experiencer verb**	**Inanimate-subject verb**	**Pronoun**
bollen<br>the ball	killen<br>the guy	sparkar<br>kick	glömmer<br>forget	träffar<br>hit	jag / mig<br>1SG
kartan<br>the map	juristen<br>the lawyer	rådfrågar<br>consult	behöver<br>need	visar<br>show	jag / mig<br>1SG
skorna<br>the shoes	döttrarna<br>the daughters	vårdade<br>nursed	fann<br>found	bar<br>carried	jag / mig<br>1SG
sjukdomen<br>the decease	direktören<br>the manager	förbannar<br>curse	fruktar<br>fear	smittar<br>infect	jag / mig<br>1SG
arbetet<br>the work	läraren<br>the teacher	berömmer<br>praise	uppskattar<br>appreciate	sysselsätter<br>engage	jag / mig<br>1SG
träningen<br>the workout	kompisen<br>the friend	tackar<br>thank	värdesätter<br>value	tilltalar<br>appeal	jag / mig<br>1SG
förslaget<br>the proposal	politikern<br>the politician	försvarar<br>defend	förknippar<br>associate	upprör<br>upset	jag / mig<br>1SG
restaurangen<br>the restaurant	mäklaren<br>the broker	rekommenderar<br>recommend	betraktar<br>regard	kompenserar<br>compensate	jag / mig<br>1SG
filmen<br>the movie	vännen<br>the friend	hyllar<br>celebrate	gillar<br>like	berör<br>affect	du / dig<br>2SG
tröjan<br>the sweater	tjejen<br>the girl	betalar<br>pay	hittar<br>find	passar<br>fit	du / dig<br>2SG
ordern<br>the order	soldaten<br>the soldier	trotsar<br>defy	uppfattar<br>perceive	vägleder<br>guide	du / dig<br>2SG
frågan<br>the question	patienten<br>the patient	besvarade<br>answered	missförstod<br>misunderstood	engagerade<br>engaged	du / dig<br>2SG
boken<br>the book	författaren<br>the writer	nämnde<br>mentioned	misstolkade<br>misinterpreted	citerade<br>quoted	du / dig<br>2SG
klänningen<br>the dress	mannen<br>the man	väljer<br>choose	ogillar<br>dislike	klär<br>suit	du / dig<br>2SG
händelsen<br>the event	offret<br>the victim	beskrev<br>described	förväxlade<br>confused	avskräckte<br>deterred	du / dig<br>2SG
solen<br>the sun	pojken<br>the boy	undvek<br>avoided	älskade<br>loved	värmde<br>heated	du / dig<br>2SG

bilen<br>the car	kvinnan<br>the woman	övergav<br>abandoned	såg<br>saw	mötte<br>met	vi / oss<br>1PL
skadorna<br>the damages	ungdomarna<br>the youngsters	rapporterade<br>reported	underskattade<br>underestimated	begränsade<br>confined	vi / oss<br>1PL
felet<br>the error	läkaren<br>the doctor	anmälde<br>reported	upptäckte<br>discovered	stoppade<br>stopped	vi / oss<br>1PL
pengarna<br>the money	forskarna<br>the scientists	samlar<br>collect	värdesätter<br>value	lockar<br>appeal to	vi / oss<br>1PL
mötena<br>the meetings	medlemmarna<br>the members	sammankallar<br>convene	övervakar<br>monitor	inspirerar<br>inspire	vi / oss<br>1PL
maten<br>the food	personalen<br>the staff	förberedde<br>prepared	avskydde<br>detested	mättade<br>satisfied	vi / oss<br>1PL
romanen<br>the novel	polisen<br>the police	uppmärksammar<br>notice	beundrar<br>admire	varnar<br>warn	vi / oss<br>1PL
politiken<br>the politician	presidenten<br>the president	klandrar<br>blame	jämför<br>compare	angår<br>concern	vi / oss<br>1PL
beslutet<br>the decision	chefen<br>the manager	kritiserade<br>critizised	respekterade<br>respected	irriterade<br>annoyed	ni / er<br>2PL
paketet<br>the package	ledaren<br>the leader	överlämnade<br>submitted	återfann<br>found again	nådde<br>reached	ni / er<br>2PL
båtarna<br>the boats	väktarna<br>the guards	utrustar<br>equip	hör<br>hear	lämnar<br>leave	ni / er<br>2PL
stugan<br>the cabin	kusinen<br>the cousin	besökte<br>visited	återsåg<br>met again	huserade<br>housed	ni / er<br>2PL
avtalet<br>the agreement	studenten<br>the student	godkände<br>approved	granskade<br>reviewed	gynnade<br>was beneficial to	ni / er<br>2PL
planen<br>the plan	flickan<br>the girl	underkände<br>failed	genomskådade<br>saw through	oroade<br>worried	ni / er<br>2PL
kontoret<br>the office	ministern<br>the minister	uppsöker<br>seek	avlyssnar<br>listen in on	stimulerar<br>stimulate	ni / er<br>2PL
låten<br>the song	journalisten<br>the reporter	utsåg<br>nominated	upplevde<br>experienced	påminde<br>reminded of	ni / er<br>2PL

5 By-participant and by-item RT differences

Figure S6 illustrates average length corrected RTs (available at https://osf.io/rw5nf/) from the self-paced reading experiment, differentiated by condition, participants and items.

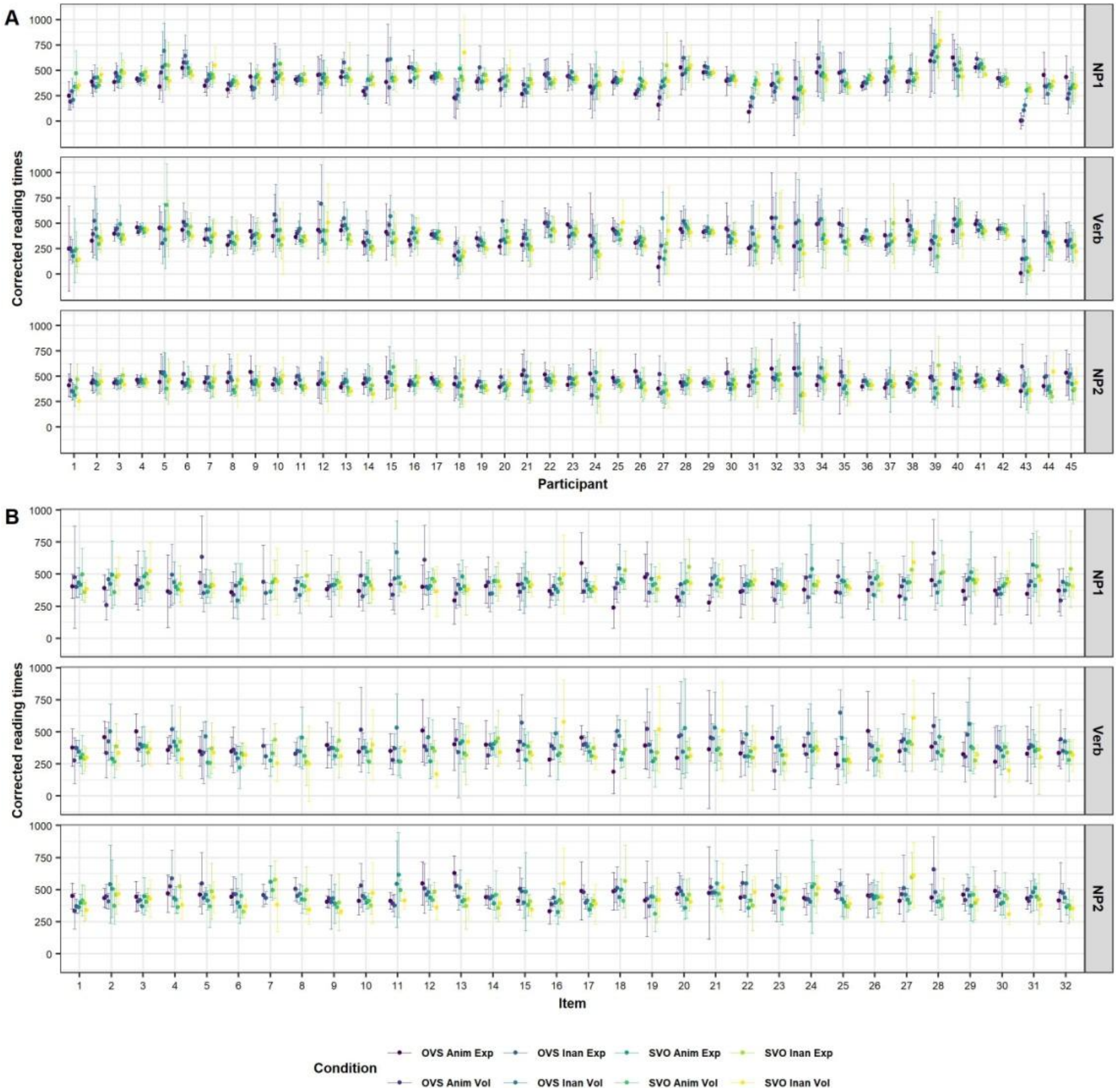

Supplementary Figure 6. RTs differentiated by conditions and participants (panel A) and items (Panel B).

6 Frequentist analyses of RT data

Here, we present frequentist analyses of the reading time data, analyzed with linear mixed effects modeling (LMM), as implemented in the R package *lme4* (Bates et al., 2015). Degrees of freedom for the calculation *p*-values in the LMMs were calculated on the basis of Welch-Satterthwaite approximation, as implemented in the *lmerTest()* package (Kuznetsova et al., 2014). Nested model comparisons were conducted on the basis of likelihood ratio tests, using the *anova()* function in R. The R script with all analyses is available at https://osf.io/rw5nf/.

6.1 Effects of linguistic cues

Analyses of the effect of linguistic cues on RTs in each region paralleled the Bayesian analyses presented in the main text. The analysis of the NP1 region contained fixed effects for object animacy (sum-coded: .5 = animate vs. -.5 = inanimate), constituent order (sum-coded: .5 = SVO vs. -.5 = OVS), and the animacy × order interaction. The analysis of the verb and NP2 region contained fixed effects for object animacy (same coding as for NP1), constituent order (same coding as for NP1), and verb (sum-coded: .5 = experiencer vs. -.5 = volitional), as well as the full factorial interactions. All models also included by-participant random intercepts and slopes for all predictors in the analysis. The results of these analyses are shown in Supplementary Table S10.

Supplementary Table S10. Results of the frequentist linear mixed-effects regressions of region-averaged length-corrected RTs investigating the effects of linguistic cues over the NP1, verb, and NP2 region. For each region, we show both the main analysis (top) and simple effects re-parameterization of the same analysis (bottom, below divider line).

Region	Parameter	$\hat{\beta}$	S.E.($\hat{\beta}$)	df	t	*p*
NP1	Intercept	-0.04	0.06	45.10	-0.65	.520
	Constituent order (*OVS* vs. *SVO*)	0.15	0.06	45.35	2.47	.017
	Object animacy (*anim.* vs. *inanim.*)	-0.04	0.03	307.56	-1.27	.205
	Order × Animacy	0.09	0.07	210.66	1.44	.151
	SVO / Animacy	0.01	0.05	289.60	0.12	.903
	OVS / Animacy	-0.09	0.05	409.99	-1.92	.056
Verb	Intercept	-0.30	0.06	45.25	-5.21	<.0001
	Constituent order (*OVS* vs. *SVO*)	-0.25	0.04	54.83	-6.19	<.0001
	Object animacy (*anim.* vs. *inanim.*)	-0.06	0.04	572.49	-1.62	.106
	Verb (*volitional* vs. *experiencer*)	-0.01	0.04	78.60	-0.24	.813
	Order × Animacy	0.16	0.08	115.63	1.94	.055
	Order × Verb	-0.02	0.08	139.59	-0.21	.836
	Animacy × Verb	-0.10	0.08	407.07	-1.33	.183
	Order × Animacy × Verb	0.12	0.16	132.94	0.75	.452
	SVO & Volitional / Animacy	0.04	0.08	930.86	0.46	.645
	SVO & Experiencer / Animacy	-0.01	0.08	136.65	-0.06	.949
	OVS & Volitional / Animacy	-0.06	0.08	125.45	-0.77	.446
	OVS & Experiencer / Animacy	-0.22	0.08	92.62	-2.61	.011
NP2	Intercept	0.08	0.02	50.69	3.95	<.0001
	Constituent order (*OVS* vs. *SVO*)	-0.17	0.03	82.96	-5.27	<.0001
	Object animacy (*anim.* vs. *inanim.*)	0.06	0.03	120.03	1.89	.062
	Verb (*volitional* vs. *experiencer*)	-0.04	0.03	221.25	-1.30	.195
	Order × Animacy	0.01	0.06	213.74	0.15	.884
	Order × Verb	0.02	0.06	1191.68	0.39	.693
	Animacy × Verb	-0.08	0.07	96.45	-1.20	.232
	Order × Animacy × Verb	0.23	0.12	605.79	1.87	.061
	SVO & Volitional / Animacy	0.05	0.06	253.05	0.76	.451
	SVO & Experiencer / Animacy	0.08	0.06	96.45	1.31	.194
	OVS & Volitional / Animacy	0.15	0.07	103.66	2.35	.021
	OVS & Experiencer / Animacy	-0.04	0.06	585.68	-0.65	.516

6.2 Effects of Bayesian surprise

Frequentist analyses of the relationships between RTs and Bayesian surprise in each region again paralleled the Bayesian analyses presented in the main text. These contained a fixed effect of standardized Bayesian surprise and by-participant random intercepts. These analyses show significant positive effects of Bayesian surprise for all three sentence regions (NP1: $\hat{\beta}_{MAP}$ = 21.24, SE = 5.12, $t(2664.70) = 4.15$, $p < .0001$; Verb: $\hat{\beta}_{MAP}$ = 2.45, SE = 0.48, $t(2670.49) = 5.06$, $p < .0001$; NP2: $\hat{\beta}_{MAP}$ = 0.14, SE = 0.03, $t(2695) = 5.63$, $p < .0001$).

6.3 The relative contribution of linguistic cues and Bayesian surprise

Frequentist model comparisons of linguistic models and Bayesian surprise models included separate nested model comparisons for each of the three sentence regions. For each region, three LMMs predicting region-based length-corrected RTs were compared: the full LMM with all linguistic cue predictors and Bayesian surprise, an LMM with the linguistic cue predictors, and an LMM with Bayesian surprise as the only predictors. All three models again included the full random effect structure from the combined analysis so that the linguistic LMM and the Bayesian surprise LMM only differed from the full model in terms of their fixed effects. The results of the two nested model comparisons—the full LMM against each of the other LMMs—are shown in Supplementary Table S11.

Supplementary Table S11. Model deviance and frequentist model comparisons, done on the basis of likelihood ratio tests, comparing full, linguistic and Bayesian surprise models, fitted on within-region RTs.

**NP1**	**Model Deviance**				
	Linguistic	Bayesian surprise		Full	
	6819.49	6822.36		6818.01	
	**ANOVA**				
			χ^2	df	*p*
	Full vs. linguistic		1.49	1	.223
	Full vs. Bayesian surprise		4.36	3	.225
**Verb**	**Model Deviance**				
	Linguistic	Bayesian surprise		Full	
	7742.67	7762.69		7741.79	
	**ANOVA**				
			χ^2	df	*p*
	Full vs. linguistic		0.88	1	.349
	Full vs. Bayesian surprise		20.90	7	.004
**NP2**	**Model Deviance**				
	Linguistic	Bayesian surprise		Full	
	6622.30	6633.10		6621.62	
	**ANOVA**				
			χ^2	df	*p*
	Full vs. linguistic		0.68	1	.411
	Full vs. Bayesian surprise		11.47	7	.120

7 The relative contribution of Bayesian surprise and word surprisal

In addition to the analyses reported in the main text, we also compared the effects of Bayesian surprise to those of word surprisal. Models of word surprisal have been the focus of the majority of previous work in computational psycholinguistics (e.g., Boston et al., 2011; Demberg & Keller, 2008; Frank & Bod, 2011; Hale, 2001; Levy, 2008, 2011), and previous evaluations have found robust effects of word surprisal on word-by-word reading times (see also Brothers & Kuperberg, 2021; Linzen & Jaeger, 2014; Smith & Levy, 2013; van Schijndel & Schuler, 2015).

Word surprisal was estimated with the GPT-2 transformer language model (Radford et al. 2019), trained for Swedish using the Swedish Blog Corpus (Östling & Wirén, 2013).[2] GPT-2 is a latent-structure inducing deep neural network, used here to predict words based on the preceding sentence context. Figure S7 illustrates the relationship between uncorrected reading times in the self-paced reading experiment and word surprisal, differentiated by sentence regions (NP1, verb, and NP2). As would be expected on previous work, we find strong positive effects of word-level surprisal on word RTs for the NP1 and verb regions. For the NP2 region, however, we observe an unexpected negative relationship.

This unexpected pattern on the NP2 region is likely due to specific properties of Swedish, and the way our model of word-level surprisal is affected by them. Word surprisal is concerned with the expectation of a specific word form given the previous context of words. At NP2, this more specifically involves the expectation of either a noun (SVO sentences) or a subject pronoun (OVS sentences) following the sentence verb. Since Swedish is a verb-second language, both of these

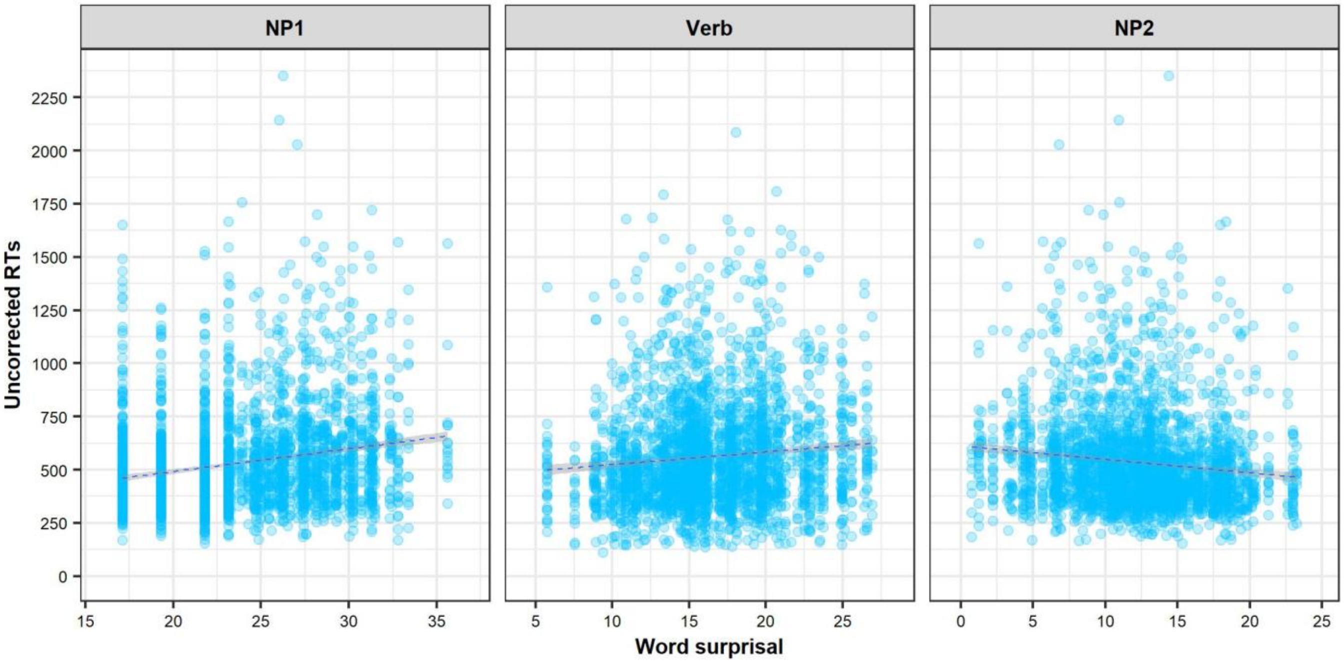

Supplementary Figure 7. Uncorrected RTs in each sentence region as function of word surprisal.

[2] We are deeply grateful to Murathan Kurfalı for fitting this model for us and providing us with word surprisal estimates for the stimuli in our experiment. We are also grateful for thoughtful input and advice from Robert Östling and Johan Sjons.

structures are common. In particular, in adverbial-initial transitive sentences, subject pronouns follow the verb. Since such sentences are quite frequent in Swedish (16.5% of the sentences in our corpus are adverbial-initial), a post-verbal subject pronoun is lower in word surprisal (mean surprisal = 9.17) than a post-verbal noun (mean surprisal = 15.45). NP2 word surprisal therefore makes the wrong predictions for reading times. This leads to the *negative* relationship between word surprisal and NP2 RTs seen in Figure S7.

7.1 Model comparison

Paralleling the model comparisons between the linguistic and Bayesian surprise LMMs, we performed separate model comparisons for the three sentence regions, comparing models with predictors for (i) Bayesian surprise and (ii) word surprisal. Both models again contained the same random effects structure, consisting of a by-participant random intercept and a slope for word surprisal. In contrast to earlier analyses, these models predicted reading times that were uncorrected for length. This decision was made since surprisal effects have traditionally been evaluated against uncorrected reading times. Model comparisons were again based on the LOOIC. In the next section, we also report model comparisons based on likelihood ratio tests.

Table S12 summarizes the results. For all three sentence regions, the Bayesian surprise LMM yields better predictive accuracy than the word surprisal LMM (lower LOOIC). This difference is large for both the verb region and the NP2 region. For the latter, the difference in predictive accuracy falls outside of the 99% interval. These results provide strong evidence—at least for the types of stimuli here—that the effects of Bayesian surprise are not reducible to the effects of word-level surprisal (as estimated here).

This is likely due to a difference in the type of expectations that word surprisal and our measure of Bayesian surprise captures. Word surprisal is concerned with the expectation of a specific word form given the previous context of words. In contrast, our measure of Bayesian surprise estimates the change in expectations for a particular argument interpretation based on the currently available cues with respect to previously presented cues, in a context that is limited to Swedish transitive clauses. Since this measure takes into account the high baseline probability for a subject-initial word order, subject case-marking on an argument NP following an initial argument NP and a verb is highly unexpected. The rational model of argument interpretation therefore correctly predicts shorter reading times in sentences with post-verbal subject pronouns: as shown in Figure 5 in the main text, Bayesian surprise is positively correlated to NP2 RTs.

Table S12. Out-of-sample predictive accuracy of the word surprisal and Bayesian surprise LMMs for each sentence region. We compare models in terms of leave-one-out cross-validated (LOO) log predictive density ($elpd_{LOO}$), specifically the difference ΔLOOIC between LMMs in the LOO information criterion (LOOIC = -2 * $elpd_{LOO}$). Confidence intervals for the differences are 2.5 standard errors below and above each difference. A confidence interval excluding zero is considered as evidence for a difference in predictive accuracy between the models at hand, and highlighted by gray shading.

**Constituent**	**LOOIC**		**LOOIC differences (ΔLOOIC)**			
	Bayesian surprise	Word surprisal	Estimate	S.E.	Lower	Upper
**NP1**	6530.94	6533.52	2.58	2.93	-4.74	9.90
**Verb**	7501.53	7527.75	26.22	10.81	-0.81	53.26
**NP2**	6469.78	6499.46	29.68	10.95	2.29	57.07

7.2 Frequentist analysis

Frequentist comparisons of Bayesian surprise LMMs and word surprisal LMMs paralleled model comparisons conducted on the Bayesian LMMs, presented in §7.1. Similar to the frequentist comparisons presented in §6.3, separate nested model comparisons were conducted for the three sentence regions, comparing LMMs with predictors for (i) both Bayesian surprise and word surprisal, (ii) only Bayesian surprise, and (iii) only word surprisal. All three LMMs again contained the same random effects structure, consisting of a by-participant random intercept and a slope for word surprisal. Again, the models predicted RTs that were uncorrected for length. The results of these comparisons are shown in Table S13.

Supplementary Table S13. Model deviance and frequentist model comparisons, done on the basis of likelihood ratio tests, comparing full, Bayesian surprise and word surprisal models, fitted on within-region RTs.

**NP1**	**Model Deviance**			
	Bayesian surprise	word surprisal	Full	
	6599.80	6605.55	6599.25	
	**ANOVA**			
		χ^2	df	*p*
	Full vs. Bayesian surprise	0.55	1	0.459
	Full vs. word surprisal	6.29	1	.012
**Verb**	**Model Deviance**			
	Bayesian surprise	word surprisal	Full	
	7548.10	7574..23	7547.80	
	**ANOVA**			
		χ^2	df	*p*
	Full vs. Bayesian surprise	0.29	1	.560
	Full vs. word surprisal	26.43	1	<.0001
**NP2**	**Model Deviance**			
	Bayesian surprise	word surprisal	Full	
	6457.90	6487.73	6455.71	
	**ANOVA**			
		χ^2	df	*p*
	Full vs. Bayesian surprise	2.19	1	.139
	Full vs. word surprisal	32.02	1	<.0001

References

Bates, D., Maechler, M., Bolker, B. & Walker, S. (2015). Fitting Linear Mixed-Effects Models Using lme4. *J. Stat. Softw*. 67, 1-48. doi:10.18637/jss.v067.i01.

Boston, M. F., Hale, J. T., Vasishth, S., and Kliegl, R. (2011). Parallel processing and sentence comprehension difficulty. *Lang. Cogn. Process. 26,* 301–349. doi:10.1080/01690965.2010.492228.

Brothers, T., and Kuperberg, G. R. (2021). Word predictability effects are linear, not logarithmic: Implications for probabilistic models of sentence comprehension. *J. Mem. Lang.* 116, 104174. doi:10.1016/j.jml.2020.104174.

Bürkner, P.-C. (2017). brms: An R Package for Bayesian Multilevel Models Using Stan. *J. Stat. Softw*. 80, 1–28. doi:10.18637/jss.v080.i01.

Bürkner, P.-C. (2018). Advanced Bayesian Multilevel Modeling with the R Package brms. *R J. Stat. Softw*. 10, 395–411. doi:10.32614/RJ-2018-017.

Dell, G. S., and Chang, F. (2014). The P-chain: relating sentence production and its disorders to comprehension and acquisition. *Philos. Trans. R. Soc. B Biol. Sci.* 369, 20120394. doi:10.1098/rstb.2012.0394.

Demberg, V., and Keller, F. (2008). Data from eye-tracking corpora as evidence for theories of syntactic processing complexity. *Cognition* 109, 193–210. doi:10.1016/j.cognition.2008.07.008.

Dowty, D. (1991). Thematic Protoroles-Roles and Argument Selection. *Language*, 67.

Einarsson, J. (1976a). *Talbankens skriftspråkskonkordans*. Unpublished manuscript, Department of Scandinavian Languages, Lund University, Lund, Sweden.

Einarsson, J. (1976b). *Talbankens talspråkskonkordans*. Unpublished manuscript, Department of Scandinavian Languages, Lund University, Lund, Sweden.

Frank, S. L., and Bod, R. (2011). Insensitivity of the Human Sentence-Processing System to Hierarchical Structure. *Psychol. Sci.* 22, 829–834. doi:10.1177/0956797611409589.

Gelman, A. (2006). Prior distributions for variance parameters in hierarchical models (comment on article by Browne and Draper). *Bayesian Anal.* 1, 515–534. doi:10.1214/06-BA117A.

Gelman, A., Jakulin, A., Pittau, M. G., and Su, Y.-S. (2008). A weakly informative default prior distribution for logistic and other regression models. *Ann. Appl. Stat.* 2, 1360–1383. doi:10.1214/08-AOAS191.

Gibson, E., Piantadosi, S. T., Brink, K., Bergen, L., Lim, E., & Saxe, R. (2013). A noisy-channel account of crosslinguistic word-order variation. *Psychological Science*, *24*(7), 1079–1088. https://doi.org/10.1177/0956797612463705

Gustafson-Capková, S., & Hartmann, B. (2006). *Manual of the Stockholm Umeå Corpus version 2.0*. Unpublished manuscript, Department of Linguistics, Stockholm University, Stockholm, Sweden.

Hale, J. (2001). "A Probabilistic Earley Parser as a Psycholinguistic Model," in *Proceedings of NAACL*, 159–166.

Heinat, F. (2010). Long object shift and reflexives. Nord. J. Linguist. 33, 67. doi:10.1017/S0332586510000053.

Kuperberg, G. R., and Jaeger, T. F. (2016). What do we mean by prediction in language comprehension? *Lang. Cogn. Neurosci.* 31, 32–59. doi:10.1080/23273798.2015.1102299.

Kuznetsova A, Brockhoff P. B., Christensen R. H. B. (2017). lmerTest Package: Tests in Linear Mixed Effects Models. *J. Stat. Softw.* 82, 1-26. doi: 10.18637/jss.v082.i13.

König, E., & Lezius, W. (2003). *The TIGER language - A Description Language for Syntax Graphs. Formal Definition*. Unpublished manuscript, University of Stuttgart, Stuttgart, Germany.

König, E., Lezius, W., & Voorman, H. (2003). *TIGERSearch 2.1*. Unpublished manuscript, University of Stuttgart, Stuttgart, Germany)

Levy, R. (2008). Expectation-based syntactic comprehension. *Cognition* 106, 1126–1177. doi:10.1016/j.cognition.2007.05.006.

Levy, R. (2011). “Integrating surprisal and uncertain-input models in online sentence comprehension: formal techniques and empirical results, ” in *Proceedings of the 49th Annual Meeting of the Association for Computational Linguistics: Human Language Technologies-Volume 1* (Association for Computational Linguistics), 1055–1065.

Linzen, T., and Jaeger, T. F. (2014). “Investigating the role of entropy in sentence processing, ” in *Proceedings of the Fifth Workshop on Cognitive Modeling and Computational Linguistics*, 10–18.

Hörberg, T. (2016). *Probabilistic and Prominence-driven Incremental Argument Interpretation in Swedish* (Ph.D. thesis). Department of Linguistics, Stockholm University, Stockholm, Sweden.

Hörberg, T. (2018). Functional motivations behind direct object fronting in written Swedish: A corpus-distributional account. *Glossa* 3, 81. doi:10.5334/gjgl.502.

MacDonald, M. C. (2013). How language production shapes language form and comprehension. *Front. Psychol.* 4. doi:10.3389/fpsyg.2013.00226.

Westman, M. (1974). *Bruksprosa*. Lund: Liber Läromedel.

Primus, B. (2012). Animacy, Generalized Semantic Roles, and Differential Object Marking. In M. Lamers & P. de Swart (Eds.), *Case, Word Order and Prominence* (Vol. 40, pp. 65–90). Dordrecht: Springer Netherlands.

R Core Team (2020). *R: A Language and Environment for Statistical Computing*. Vienna, Austria: R Foundation for Statistical Computing, available at: https://www.R-project.org/.

Radford, A., Wu, J., Child, R., Luan, D., Amodei, D., and Sutskever, I. (2019). Language Models are Unsupervised Multitask Learners. *OpenAI Blog* 1.

Smith, N. J., and Levy, R. (2013). The effect of word predictability on reading time is logarithmic. *Cognition* 128, 302–319. doi:10.1016/j.cognition.2013.02.013.Stan Development Team (2017). *Stan Modeling Language: User’s Guide and Reference Manual*. Available at: mc-stan.org.

van Schijndel, M., & Schuler, W. (2015). “Hierarchic syntax improves reading time prediction,” in *Proceedings of the 2015 Conference of the North American Chapter of the Association for Computational Linguistics: Human Language Technologies*, 1597-1605.

Vehtari, A., Gelman, A., and Gabry, J. (2017). Practical Bayesian model evaluation using leave-one-out cross-validation and WAIC. *Stat. Comput.* 27, 1413–1432. doi:10.1007/s11222-016-9696-4.

Östling, R., and Wirén, M. (2013). “Compounding in a Swedish Blog Corpus,” in *Computer mediated discourse across languages Stockholm Studies in Modern Philology.*, eds. L. Á. López, C. S. Brylla, and P. Shaw, (Stockholm: Stockholm University), 45–63.
